# Strategic restoration of global inland peatlands can maximize local and global co‐benefits

**DOI:** 10.1002/eap.70300

**Published:** 2026-09-08

**Authors:** Emily A. Ury, Zhen Zhang, Etienne Fluet‐Chouinard, Stavroula S. Sartzetakis, Tianyi Sun, Brian Buma

**Affiliations:** ^1^ Environmental Defense Fund New York New York USA; ^2^ National Tibetan Plateau Data Center (TPDC), State Key Laboratory of Tibetan Plateau Earth System, Environment and Resource (TPESER), Institute of Tibetan Plateau Research, Chinese Academy of Sciences Beijing People's Republic of China; ^3^ Earth System Science Division Pacific Northwest National Laboratory Richland Washington USA; ^4^ Present address: Colgate University Hamilton New York USA

**Keywords:** ecosystem services, greenhouse gas, methane, peatlands, restoration, wetlands

## Abstract

Global‐scale peatland restoration holds large potential for carbon sequestration; however, the peatland carbon balance is not often considered alongside co‐benefits in restoration prioritization. Here, we present a spatial optimization framework for inland peatland restoration to minimize greenhouse gas (GHG) emissions and maximize peatland co‐benefits, such as flood mitigation and water quality improvements. We find strategic restoration of 30% of the world's drained inland peatlands, targeting areas with the greatest potential for GHG emissions reductions, would sequester three times more GHGs (0.91 ± 0.17 Pg CO_2_eq. year^−1^) than the same magnitude of peatland restoration implemented randomly (0.27 ± 0.11 Pg CO_2_eq. year^−1^). Peatland restoration is often driven by local needs for the many ecosystem services that peatlands provide; therefore, we also quantify the GHG balance of restoration aimed at delivering either water quality improvements or flood mitigation. Meeting a 30% global inland peatland restoration target while prioritizing water quality improvements or flood mitigation can still achieve emissions reductions of 0.34 ± 0.14 and 0.50 ± 0.19 Pg CO_2_eq. year^−1^ (62% and 44% less emissions reductions than prioritizing emissions alone), respectively, demonstrating opportunities for overlap between global GHG budgets and local environmental objectives. Finally, we use future methane (CH_4_) emissions projections to evaluate the compatibility of peatland restoration prioritization schemes with future CH_4_ emissions. We find that priority regions for near‐term GHG emissions reduction do not necessarily align with areas that would minimize future peatland CH_4_ emissions, due to increasing CH_4_ emissions by 2100. We account for uncertainty and variability in long‐term emission trajectories of peatland restoration from diverse settings, site histories, and practices by testing our optimization framework with two alternate endpoint scenarios: “recently rewetted” and “restored to intact conditions.” Our framework highlights numerous opportunities for inland peatland restoration to simultaneously achieve both near‐ and long‐term emission reductions as well as multiple co‐benefits. Broad‐scale restoration strategies can be employed in concert with planning for regional needs and site‐specific criteria to magnify the benefits of peatland restoration.

## INTRODUCTION

There is considerable interest and investment in the restoration of degraded wetlands and peatlands globally, owing to their numerous ecological benefits and ecosystem services (Andersen et al., 2017; Chimner et al., 2017; Wang et al., 2012). Peatland conservation and restoration is widely considered a “natural climate solution” (Fargione et al., 2018; Griscom et al., 2017; Humpenöder et al., 2020; Leifeld & Menichetti, 2018; Strack et al., 2022) due to peatland's potential for long‐term carbon uptake and storage (Petrescu et al., 2015). The long‐term accumulation of organic material in low‐decomposition conditions makes peatlands among the most carbon‐rich ecosystems in the world (Leifeld & Menichetti, 2018; Petrescu et al., 2015; Turetsky et al., 2015).

Drainage, conversion, and degradation of peatlands for agriculture, forestry, peat extraction, and other land uses lead to aerobic conditions and high rates of organic matter mineralization to carbon dioxide (CO_2_) (Günther et al., 2020; Nugent et al., 2018; Wilson et al., 2016), but their restoration can reestablish peatlands as stable carbon stocks and net carbon sinks (Günther et al., 2020; Neubauer & Megonigal, 2021; Qiu et al., 2022). However, the greenhouse gas (GHG) balance of peatland rewetting involves a biogeochemical trade‐off whereby restoring a shallow water table reinitiates natural methane (CH_4_) emissions that can outweigh carbon sequestration on short timeframes (He et al., 2024; Hemes et al., 2018; Koffi et al., 2020). The renewed CH_4_ production following rewetting must also be weighed against strongly reduced CO_2_ and nitrous oxide (N_2_O) emissions under restored conditions. Thus, peatland restoration aiming to reduce GHG emissions should consider the GHG balance of these three gases during pre‐ and post‐restoration periods. Spatial variability of this GHG balance is driven by climate and land use and can inform decision making for priority areas for peatland restoration. Under ambitious global restoration targets (i.e., 30% by 2030; CBD, 2022; European Commission, 2020), there is an urgent need to prioritize locations that lead to the largest immediate improvements in the carbon and GHG balance of peatlands, as well as the consideration of synergies and trade‐offs with co‐benefits.

Evaluating the trade‐off between carbon sequestration and CH_4_ emissions in peatlands inherently depends on the timeframe of analysis (Günther et al., 2020; Taillardat et al., 2020). Due to the short atmospheric lifetime of CH_4_, warming effects from CH_4_ emissions can offset peatland carbon sequestration over decadal timescales, while the cooling effect from carbon sequestration dominates over century‐long timescales (Günther et al., 2020). Typically, the global warming potential (GWP) of GHG emissions is evaluated over a 100‐year time horizon, wherein the enhanced warming effect of CH_4_ is heavily discounted by its short lifespan in the atmosphere (Ocko et al., 2017). The impact of restoration prioritization will therefore depend on the time horizon used to calculate the GHG balance. Another important time‐dependent factor affecting the GHG budget of peatland restoration is that CH_4_ emissions are expected to increase significantly due to projected changes in temperature and precipitation (Feng et al., 2022; Nisbet, 2023; Zhang et al., 2017). This will enhance CH_4_ emissions from intact and restored peatlands alike, forming a positive feedback loop with climate warming (Bao et al., 2023; Li et al., 2022; Zhang et al., 2023). Avoiding peatland restoration in areas with high future CH_4_ emissions potential deserves consideration among strategies for pursuing long‐term climate stability. Therefore, both spatial and temporal variation in peatland GHG budgets must be considered when setting priorities for strategic restoration.

Aside from carbon sequestration, peatland restoration can be pursued for water quality, flood protection, and habitat for biodiversity, among other functions (Acreman & Holden, 2013; Andersen et al., 2017; Bonn et al., 2016). Typically, restoration projects are implemented on an individual basis, driven by the need for local ecosystem service delivery and influenced by local land‐use trade‐offs and stakeholder buy‐in. There have, however, been several recent efforts to devise coordinated wetland restoration strategies at landscape scales to meet regional goals for water quality (Cheng et al., 2020; Tomscha et al., 2023; Zimmerman et al., 2019) and flood risk mitigation (Gourevitch et al., 2020). Similar coordinated approaches might be used in peatland restoration planning and incorporate the aim of maximizing the contribution to climate change mitigation. Moreover, planning peatland restoration priorities at national to global scales, as has been done for other ecosystems (Strassburg et al., 2020), can improve restoration outcomes on multiple axes but needs to be implemented in concert with location‐specific and site‐level restoration strategies. Opportunities to address multiple objectives through restoration offer a rationale for expanding investment from multiple stakeholders to spur widespread restoration efforts. However, strategic restoration strategies for peatlands and wetlands rarely consider GHG emissions and other ecosystem services simultaneously (Hambäck et al., 2023; Tanneberger et al., 2024; Widis et al., 2015).

Here, we developed a prioritization scheme for inland peatland restoration based on the net carbon and GHG balance and evaluated optimized outcomes in comparison to a random scenario, as well as against scenarios prioritizing two other key ecosystem services. The GHG balance considers the biogeochemical trade‐off between carbon sequestration and CH_4_ emissions on 20‐ and 100‐year horizons, including additional CH_4_ arising from climate change feedbacks (Hemes et al., 2018). We leverage the spatial variability in projected future emissions of drained, inland peatlands to devise restoration strategies optimized for either near‐term or long‐term emissions reductions and identify opportunities that maximize climate benefits on both timelines. Alternative prioritization scenarios, aimed at meeting multiple restoration objectives including flood mitigation and water quality improvements, are also evaluated for their climate impact. Due to data limitations, we do not construct time‐resolved peatland GHG budgets, but instead evaluate trade‐offs between instantaneous GHG balance at present and end‐of‐century under different fractions of drained peatland extent restored. Our goal is to provide perspective on the capacity for beneficial outcomes from coordinated, strategic peatland restoration rather than generate a prescriptive restoration plan competing with local‐scale considerations.

## METHODS

### Method overview

We built global maps of peatland GHG emissions by combining a peatland extent map (Melton et al., 2022) with emission estimates from several sources (Bahram et al., 2022; Temmink et al., 2022; Zhang et al., 2017). Emissions factors from drained and rewetted peatlands (IPCC, 2014) were used to estimate changes in emissions between drained and restored peatlands based on land use and climate zone. We performed Monte Carlo simulations to incorporate uncertainty in emissions estimates into spatially explicit estimates of the net GHG emissions from peatland restoration. The resulting maps were used to assess strategies for restoration, including prioritization based on net climate impact. A complete list of source information for the spatial data used in this analysis can be found in Appendix S1: Table S1.

### Global inland peatland area and land‐use change

The present‐day distribution of global peatlands was based on the model Peat‐machine learning (ML) from Melton et al. (2022), which leverages ML to overcome underestimates in areas where peatlands have been poorly mapped. Melton et al. (2022) use an inclusive definition of peatland—areas with or without vegetation that contain at least 30% dead organic material by dry mass—which is ideal for our purpose of identifying potentially restorable peatland. To identify the geographic extent and distribution of drained peatlands that could be restored, we used a reconstruction of historic wetlands (Fluet‐Chouinard et al., 2023) and assumed that historic peatland extent is proportional to the present‐day fraction of peatland to all wetland within a grid cell (following Fluet‐Chouinard et al., 2023). This peatland fraction allows us to identify areas of drained, and potentially restorable, peatland (Appendix S1: Figure S1) assuming the loss of peatland and non‐peatland wetland types are proportional to their present distribution. The map of drained peatlands was masked to non‐coastal pixels to ensure alignment with CH_4_ emissions estimates (see below), which exclude coastal wetlands. All maps were resampled and aligned to match the 0.5‐degree grid cell resolution of the wetland reconstruction (Fluet‐Chouinard et al., 2023).

### Emissions estimates across peatland states

We estimate the net climate benefit of restoration as the difference between emissions from drained and restored peatlands. We represent peatland transitions in three stages: intact, drained, and then one of two alternative restored states. As long‐term restoration pathways of peatlands are uncertain due to initial conditions, hydroclimatic conditions, and restoration practices (Mander et al., 2024), we explore two alternative endpoints described below as “recently rewetted” and “restored to intact conditions.” These two endpoints are idealized trajectories meant to capture the wide range of outcomes from widespread peatland restoration.

For the “intact” peatland state (pre‐drainage), representing peatlands that have not undergone major disturbance or drainage, estimates for CH_4_ emissions come from the LPJ‐wsl global land surface model that incorporates both top down and bottom‐up inputs to generate 0.5‐degree resolution wetland emissions estimates (Zhang et al., 2017). We applied the simulated CH_4_ flux rates for wetlands to our intact peatland cover to get peatland emissions. Emissions for N_2_O come from a global meta‐analysis (Bahram et al., 2022); the original data were reanalyzed to find the mean and SD for intact peatlands based on climate zones (boreal, temperate, and tropical; see Appendix S1: Figure S2a) derived from the Köppen‐Geiger climate classification (Beck et al., 2023). Modern day carbon sequestration rates were used as a proxy for net CO_2_ emissions; climate zone mean and SD are from a meta‐analysis of global peatlands (Temmink et al., 2022). We assumed carbon fluxes only occur between the peatland surface and the atmosphere and ignored lateral fluxes of carbon to and from peatlands in our GHG budgets.

Emissions factors for CO_2_, CH_4_, and N_2_O for drained peatlands are adapted from the Intergovernmental Panel on Climate Change (IPCC) Wetlands Supplement (IPCC, 2014). The IPCC Wetlands Supplement (IPCC, 2014) represents the most comprehensive synthesis of emissions factors from drained peatlands with specific reference to the land use following drainage. Land use following drainage is a good indicator of the drainage depth and level of peat degradation (e.g., for cultivation vs. peat extraction), which are important drivers of GHG fluxes (Mander et al., 2024). To best leverage emission factors, we combined land‐use types into the following broad groupings: agriculture, forestry, peat extraction, and rice cultivation (see Appendix S1: Figure S3, Tables S2 and S3 for aggregated emissions factors and land‐use groupings). These classes were specifically chosen to align with land‐use categories from the historic wetland reconstruction from Fluet‐Chouinard et al. (2023) that distinguishes land‐use type following peatland drainage. Drainage to urban land use was omitted from the analysis owing to lack of emissions data and the limited capacity for restoring peatlands following conversion to urban land uses. Methane emissions from tropical peat extraction (<15 km^2^ globally) are not covered in the IPCC Wetlands Supplement, but for the purpose of our analysis is assumed to be the same as emissions in temperate zones, following Günther et al. (2020). As emissions factors for rice cultivation in temperate climates are also not reported by the IPCC, we use alternative emissions factors from Qian et al. (2023) for CH_4_ and N_2_O, recalculated over our climate zones. To fill the gap for CO_2_ emissions from temperate rice cultivation, we scaled down the emissions from tropical rice cultivation, as temperate GHG emissions are generally lower than tropical (Wang et al., 2018). We found the relative proportion of emissions between temperate and tropical zones in published literature for CH_4_ (0.81) and N_2_O (0.77) from Qian et al. (2023) and used the average (0.79) as a scaling factor to estimate temperate rice CO_2_ emissions from the tropical rice CO_2_ emission factor reported by the IPCC (2014).

Two different approaches were used to estimate GHG emissions following peatland restoration. In the first approach, we use emissions from the IPCC Wetlands Supplement for rewetted peatlands (IPCC, 2014), following the same procedure as described for drained peatlands. The IPCC emissions factors represent the best available estimates of emissions from rewetted peatlands; however, they are biased toward recently rewetted peatlands and do not capture the dynamics or long‐term fate of emissions following rewetting (Bianchi et al., 2021; Kalhori et al., 2024; Qiu et al., 2021). Without the ability to sufficiently describe the full trajectory of peatland emissions following rewetting, we make the assumption that these emissions will converge toward emissions from intact peatlands within the same geographic context (Li et al., 2022). We therefore treat the rewetted peatland emissions factors from the IPCC as an intermediate endpoint representing “recently rewetted” peatlands and contrast this with a second endpoint representing peatlands “restored to intact conditions.” This second approach simply applies the emissions factors from intact peatlands (described above) as those following peatland restoration. This approach assumes that sufficient lag time has elapsed for the peatland to return to pre‐drained ecological conditions. While there is evidence that some restored peatlands do not return to their initial intact conditions, even after multiple decades (Bianchi et al., 2021; Loisel & Gallego‐Sala, 2022), other studies suggest a return to similar function is possible after 7–17 years (coastal wetlands) (O'Connor et al., 2020), or 20–64 years in wetlands following agricultural land use (Tangen & Bansal, 2020). We therefore assume that the recovery trajectory for most restored peatlands will revert to emissions similar to an intact peatland in the same geographic context within a matter of decades.

### Calculating net emissions change from peatland restoration

Due to the uncertainty of the long‐term endpoint of restored peatlands and their potential GHG emissions, we estimated restored emission using both the “recently rewetted” and “restored to intact” states as endpoints. The IPCC does not disaggregate emissions from rewetted peatlands by former land use, leading us to use the same emission rates for all rewetted peatlands in a given climate zone. Total emissions following restoration (*E*_restored_) for each endpoint are calculated as follows:

(1) $$E_{\text{restored},20}={\mathrm{FCO}}_{2}\mathrm{eq}.\times {\text{Area}}_{\text{drained}}=\left({\mathrm{FCO}}_{2}+96\;{\mathrm{FCH}}_{4}+250\;{\mathrm{FNO}}_{2}\right)\times {\text{Area}}_{\text{drained}},$$

(2) $$E_{\text{restored},100}={\mathrm{FCO}}_{2}\mathrm{eq}.\times {\text{Area}}_{\text{drained}}=\left({\mathrm{FCO}}_{2}+45\;{\mathrm{FCH}}_{4}+270\;{\mathrm{FNO}}_{2}\right)\times {\text{Area}}_{\text{drained}},$$

where FCO_2_eq. is the total GHG flux in terms of CO_2_ equivalents as determined using the sustained‐flux GWPs over 20‐ (SGWP_20_) and 100‐year (SGWP_100_) time horizons (Neubauer & Megonigal, 2015). We acknowledge there are some drawbacks of using SGWPs rather than other metrics of CO_2_ equivalence or time integrated radiative forcing (Ma et al., 2024), but it adequately represents this emissions source and is the most feasible option for the spatially resolved analysis performed here (Frolking & Roulet, 2007). The flux of each individual GHG (FCO_2_, FCH_4_, and FN_2_O) is scaled by the relative warming potential for the respective time horizon, as shown in the right‐hand sides of Equations (1) and (2). Area_drained_ refers to the total area of drained peatland within each grid cell.

The net change in emissions following restoration (*E*_net_) must account for the change in emissions from the drained state; therefore, the emissions from drained peatlands are subtracted from the emissions potential following restoration. Emissions from all drained peatlands globally are calculated as the sum over all grid cells, *j*, of the difference between emissions following restoration and emissions from the drained state:

(3) $$E_{\mathrm{net},\text{global}}=\Sigma {\left(E_{\text{restored}}-\Sigma F_{\text{drained},i}\times {\text{Area}}_{\text{drained},i}\right)}_{j},$$

where emissions from drained peatlands are the sum of the area‐normalized GHG flux (*F*_drained_) from each drained land‐use type, *i*, within that grid cell (agriculture, forestry, peat extraction, or rice cultivation). *E*_net_ <0 indicates a GHG sink whereas positive net emissions indicate a GHG source.

### Restoration prioritization for climate outcomes

In each scenario prioritizing one or multiple objectives, we examine both strategic and random approaches for prioritizing peatland restoration. For “climate‐first” strategic peatland restoration, peatlands are restored in the order from lowest (i.e., a reduction in the net GHG balance) to highest emissions potential in terms of SGWP. The random scenario is essentially a null case representing no strategic action. We recognize that, in reality, restoration planning is not random but rather carried out in an “ad hoc” fashion, dictated by external factors such as land availability, opportunity, interest, and financial factors (Glenk & Martin‐Ortega, 2018). We represent ad hoc restoration with a scenario that is randomized with respect to net emissions, the “random” scenario.

We generated restoration prioritization scenarios for all peatlands drained since 1700, as well as for a subset of only peatland drained post‐2010. This separation into two prioritizations circumvents our inability to account for “time since drainage” with emission factors. Recently drained peatlands are prime candidates for restoration due to the initial pulse of carbon loss in the years following drainage (Ma et al., 2022; Qiu et al., 2021), which yields a larger benefit from avoided emissions. Future studies quantifying avoided carbon emissions would help improve strategies for “climate‐smart” wetland and peatland restoration but are beyond the scope of the present work.

### Multiobjective restoration scenarios

In addition to the “climate‐first” scenario, we assess two scenarios that prioritize restoration to maximize other ecosystem services: water quality improvements and flood mitigation. For the potential of restoration to yield water quality improvements, we focus on the role of peatlands in trapping pollutants and in particular removing nitrogen from agricultural runoff through denitrification (Cheng & Basu, 2017; Zedler, 2003). A global nitrogen fertilizer use map (Potter et al., 2010, 2012) was used as a proxy for nitrogen sources and agrochemical polluted waterways (Appendix S1: Figure S2b). This follows previous work linking fertilizer use to degradations in local water quality conditions (Beusen et al., 2022; Yu et al., 2019). For flood mitigation, fluvial flood hazard maps (Dottori et al., 2016) were used to assess the area in each 0.5‐degree grid cell that is at risk of flooding during a 100‐year storm event (Appendix S1: Figure S2c). For both ecosystem services, a multi‐objective prioritization scheme was established to first address the objective flood (“flood first”) or water quality (“nitrogen first”), then net GHG emissions reductions. Both multi‐objective restoration scenarios were run for all drained peatlands and for the subset of only peatlands drained post‐2010.

We also evaluated the future climate impact of restoration scenarios by evaluating the effect of using SGWP over a 20‐ versus 100‐year time horizon to evaluate the CO_2_ equivalents of peatland GHG emissions. The more frequently used 100‐year time horizon heavily discounts the warming potential of CH_4_ due to its relatively short atmospheric lifespan (Ocko et al., 2017), whereas the warming impact of CH_4_ is more pronounced on shorter timescales (Neubauer & Megonigal, 2015). The mischaracterization of the climate forcing effects of these gases can hinder wetland and peatland management and restoration efforts (Li et al., 2022; Worrall et al., 2009). Therefore, we primarily use the 20‐year time horizon throughout our analyses, but we demonstrate the future climate impact and the effect of the discount by comparing these two time horizons.

Finally, we consider future peatland CH_4_ emissions in 2099 simulated by LPJ‐wsl in Zhang et al. (2017) to consider long‐term climate stability as a consideration for restoration planning, which may be in opposition with near‐term climate objectives. A restoration scenario is generated to minimize future CH_4_ emissions by avoiding areas of high CH_4_ emissions growth by 2099. The simulated future CH_4_ fluxes account for the influence of climate change‐driven feedbacks, under Representative Concentration Pathway (RCP) 4.5, on CH_4_ production and change in inundation extent, but not expansion due to peatland restoration. The 2099 CH_4_ emissions projection (ECH_4 2099_) is used to determine spatial patterns of peatland CH_4_ emissions growth (Appendix S1: Figure S4), calculated as

(4) $${\mathrm{CH}}_{4}\;\text{emissions growth}={\mathrm{ECH}}_{4\;2099}-{\mathrm{ECH}}_{4\;2020}.$$

RCP 4.5 was chosen to illustrate a “middle of the road” future emissions scenario (Meinshausen et al., 2020; Riahi et al., 2017), wherein global CH_4_ from all wetlands will increase from 172 to 255 Tg CH_4_ year^−1^ by 2100 (Zhang et al., 2017). These future simulations capture projected increases in peatland CH_4_ emissions from a projection of meteorology, inundation extent, and rising atmospheric CO_2_ concentrations, but ignore the biogeochemical effects of sulfate and nitrogen deposition on peatland CH_4_ production, and the effect of climate change on future N_2_O or CO_2_ fluxes from peatlands. Changes in the fluxes of N_2_O and CO_2_ may partially or completely compensate for the additional CH_4_ or further contribute to the net GWP, and this unknown should be the focus of further investigation.

### Statistical analyses

Monte Carlo simulations were used to estimate uncertainty associated with GHG emissions using the mean and SD of emission factors in each climate zone and land‐use combination to generate random parameter sets assuming a normal distribution around the mean. For N_2_O and CO_2_ from intact peatlands (Bahram et al., 2022; Temmink et al., 2022), the SD was calculated directly from the source data (see Appendix S1: Table S1). For CH_4_, the SD was estimated as 20% of the local modeled emission value based on approximate model variance reported. SDs for the IPCC emissions factors were calculated from the supplied mean, sample size, and the 95% CI. In some instances, the SD could not be estimated in this way due to insufficient or unreported sample size, leading us to substitute the SD from the most similar emission factor in these cases (see available code for specific substitutions). We conducted 500 Monte Carlo simulations to produce the means and SDs reported in the results.

All analyses were conducted at a 0.5‐degree resolution in the R statistical computing environment (version 4.2.3) using the following packages: “raster” (Hijmans, 2023), “sf” (Pebesma, 2018), “ncdf4” (Pierce, 2023), “ggplot2” (Wickham, 2016), “cowplot” (Wilke, 2020), and “tidyverse” (Wickham et al., 2019).

## RESULTS

**FIGURE 1:**
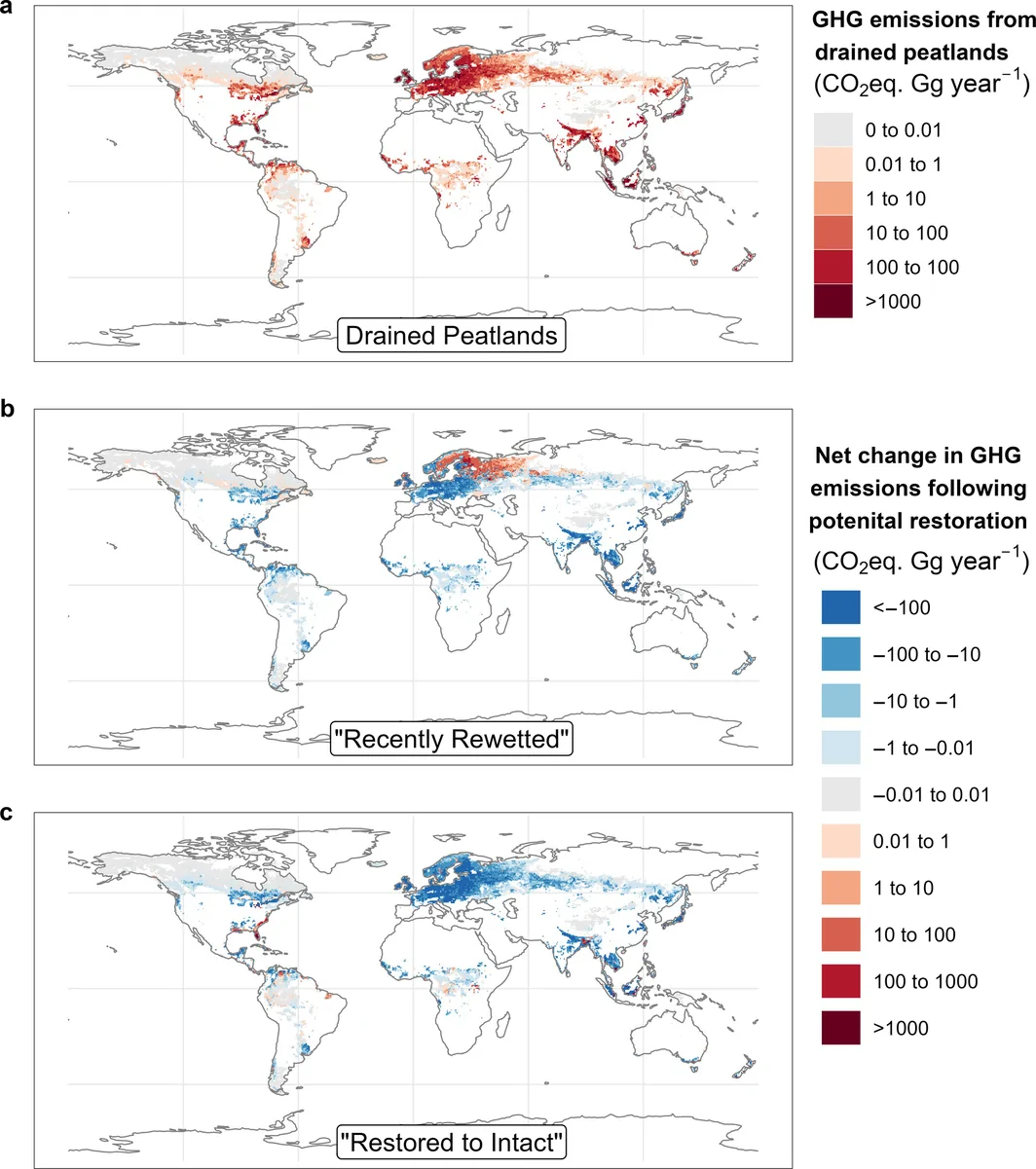
Greenhouse gas (GHG) emissions from drained peatlands and net change in emission following restoration. (a) Total GHG emissions from drained peatlands. (b, c) Change in emissions following conversion of all drained peatlands to “recently rewetted” (b) or “restored to intact” (c) conditions as the final endpoints. Blue colors indicate net negative emissions (GHG sequestration), and red colors indicate net positive emissions. Emissions are expressed as by‐pixel sums of CO_2_, CH_4_, and N_2_O, converted into CO_2_eq. using a sustained‐flux global warming potential over 20 years (SGWP20) and mapped over areas of global peatland drainage. See Appendix S1: Figure S5l (“recently rewetted”) and Figure S5p (“restored to intact”) for emissions estimates of restored peatlands prior to subtracting emissions from drained peatlands. Note that the color scale does not represent equal sized breaks in the data and grid cells with less than 1% present‐day peatland coverage have been masked out for visual clarity.

Drained peatland emissions estimates differ across land uses following drainage, but overall, drained peatlands are large contributors of GHG emissions and have a positive radiative balance due to high CO_2_ emissions, modest but not insignificant CH_4_ emissions, and N_2_O emissions (Figure 1a). Similarly to drained peatlands, hypothetically “recently rewetted” peatlands would have a positive radiative balance across the majority of potentially restorable peatlands (Appendix S1: Figure S5), owing to large CH_4_ contributions and low carbon sequestration rates. In contrast, if “restored to intact conditions,” peatlands could have either a positive (54% of potentially restorable peatland area) or negative (46%) radiative balance, depending on the balance between CH_4_ plus N_2_O emissions and CO_2_ uptake between the drained and restored states (Appendix S1: Figure S5). The net change in emissions following peatland restoration, accounting for emissions avoided from the drained state, results in a negative radiative forcing in most cases (Figure 1b,c). Restoration to “intact conditions” would lead to a median net change in emission of −31 tCO_2_eq. ha^−1^ year^−1^ (range: −96 to 2500 tCO_2_eq. ha^−1^ year^−1^), with 88% of restored peatland area resulting in net GHG emissions reductions. For “recently rewetted” peatlands, the median net change in emissions would be −39 tCO_2_eq. ha^−1^ year^−1^ (range: −104 to −14 tCO_2_eq. ha^−1^ year^−1^), and 85% of rewetted peatland area resulting in GHG emissions reductions.

We estimated emissions reductions of 1.1 ± 1.8 Pg CO_2_eq. year^−1^ (mean and SD from 500 Monte Carlo simulations) from returning all drained peatlands to “recently rewetted” conditions and 0.95 ± 0.37 Pg CO_2_eq. year^−1^ when restoring all drained peatlands to “intact conditions.” Input models for CH_4_ emissions from Zhang et al. (2017) have higher spatial resolution and lower variance than the IPCC emissions estimates, which yields more spatial specificity and narrower CIs. Therefore, we focus on the “restored to intact” endpoint in the restoration scenario results presented below.

### Optimized restoration to reduce GHG emissions

**FIGURE 2:**
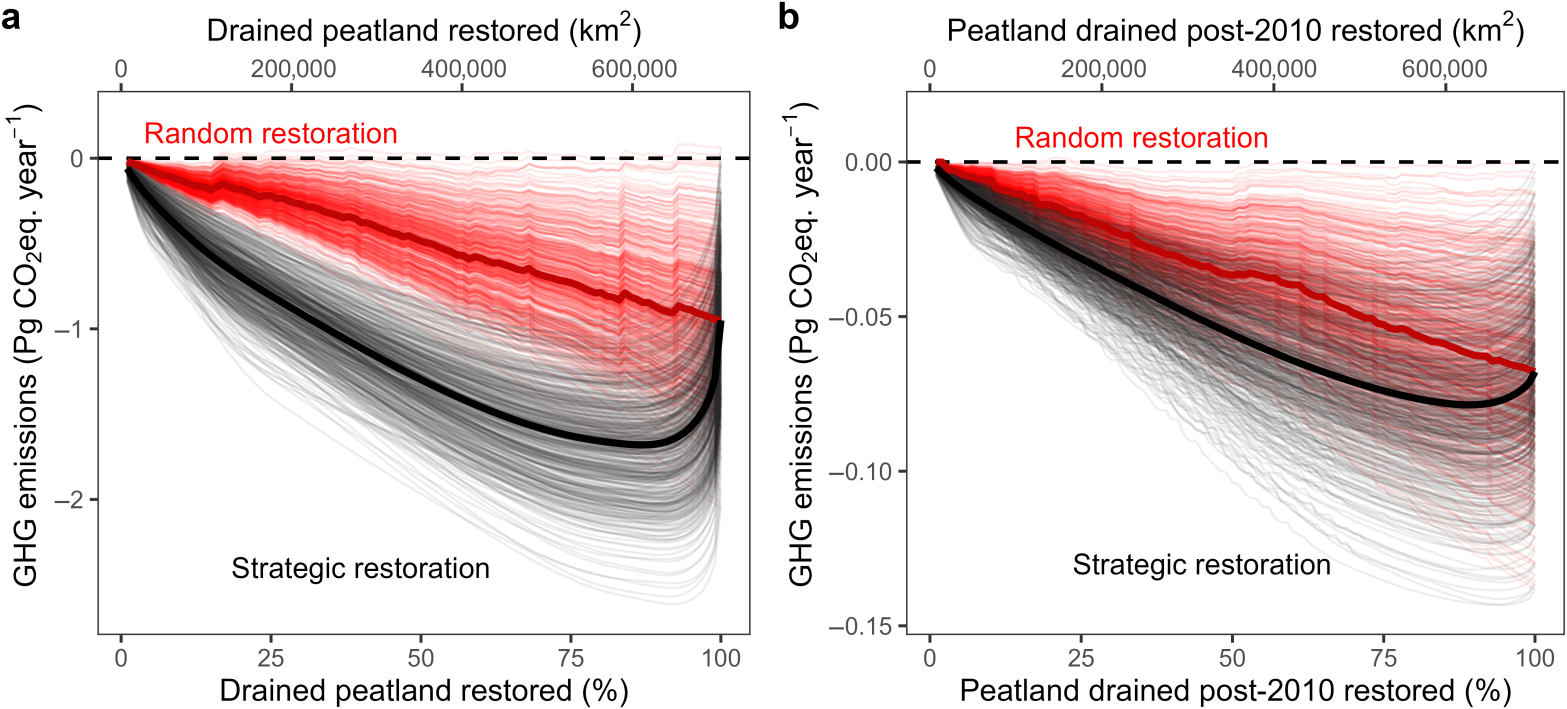
Net change in greenhouse gas (GHG) emission from restoration of drained peatlands. (a) Net change in GHG emissions in terms of CO_2_ equivalents (sustained‐flux global warming potential over 20 years [SGWP20]) for strategic (black lines) and random (red lines) restoration scenarios across 0%–100% drained peatlands restored to “intact conditions.” Each line represents one iteration of the Monte Carlo simulation, and the bold lines represent the scenario means. The variation in emissions at 100% restoration is due to uncertainty in emission as represented by the Monte Carlo simulations. The scenario for “recently rewetted” peatlands is shown in Appendix S1: Figure S7. (b) Change in GHG emissions from restoration of only peatlands drained post‐2010. A map of post‐2010 drained peatland area can be found in Appendix S1: Figure S1c.

Area‐normalized net change in emissions following restoration (Appendix S1: Figure S6) is used to build a strategic restoration scenario for prioritized peatland restoration. Strategic restoration enhances GHG emissions reductions by as much as 0.92 Pg CO_2_eq. year^−1^ relative to a random scenario (Figure 2a for “restored to intact” and Appendix S1: Figure S7 for the “recently rewetted” endpoint); this maximum benefit occurs when an optimal 68% of drained peatland area is restored (random_68%_ = 0.65 ± 0.25 Pg CO_2_eq. year^−1^, strategic_68%_ = 1.56 ± 0.27 Pg CO_2_eq. year^−1^). The cumulative benefits of peatland restoration for the climate begin to taper off after 88% drained peatland restoration is reached (Figure 2a).

When prioritizing restoration in areas with the largest net decrease in GHG emissions, restoring only 1% of the world's drained peatland could reduce emissions by 0.060 ± 0.021 Pg CO_2_eq. year^−1^. Conversely, avoiding restoration in the 12% of peatlands where restoration would lead to a net increase in emissions (areas with a potential for very high CH_4_ emissions) could also have a significant impact on reducing the carbon footprint of peatland restoration (Table 1). Restoring the 12% of peatlands with net positive emissions from restoration would cumulatively contribute 0.70 ± 0.26 Pg CO_2_eq. year^−1^ worth of GHG emissions and offset most of the emissions reductions from peatland restoration elsewhere. Restoring only the top 1% of high emissions areas following restoration could result in 0.34 ± 0.023 Pg CO_2_eq. year^−1^ of additional GHG emissions, underscoring the value of avoiding restoration in areas with substantial emissions potential. Notable areas with a high potential for increased GHG emissions following peatland restoration are found in the southeastern United States, Bangladesh, and elsewhere in tropical latitudes (red colored areas in Figure 1c).

**TABLE 1:** Net change in greenhouse gas (GHG) emissions from peatland restoration efforts of varying magnitude with data grouped by order of peatland restoration.

Drained peatland area restored (%)	Mean (SD) net change in GHG emissions (Pg CO_2_eq. year^−1^)^a^		
	Low emissions first	Random	High emissions first^b^
1	−0.060 (0.021)	−0.016 (0.004)	0.34 (0.023)
3	−0.16 (0.055)	−0.043 (0.011)	0.53 (0.027)
10	−0.42 (0.11)	−0.13 (0.038)	0.72 (0.066)
30	−0.91 (0.17)	−0.27 (0.11)	0.63 (0.12)
50	−1.3 (0.22)	−0.49 (0.18)	0.35 (0.17)
99	−1.3 (0.37)	−0.94 (0.37)	−0.89 (0.36)
100	−0.95 (0.37)	−0.95 (0.37)	−0.95 (0.37)

^a^ Calculated using sustained‐flux global warming potential over 20 years (SGWP_20_).

^b^ These estimates are derived from the same data as presented in Figure 2 with the order of prioritization reversed.

Comparing strategic and random restoration while using SGWP over a 100‐year time horizon (Appendix S1: Figure S8), we find emissions reductions of 1.65 ± 0.35 Pg CO_2_eq. year^−1^, nearly double those calculated over a 20‐year time horizon (SGWP_20_, 0.95 ± 0.37 Pg CO_2_eq. year^−1^) for restoration of all peatlands. Restoration in some temperate regions only produces high emissions reductions potential when a 20‐year time horizon is employed, whereas using 100‐year time horizons illustrates the cooling potential of tropical peatland restoration over longer timeframes (Appendix S1: Figure S8c).

Restoring recently drained peatlands (post‐2010), which are typically characterized by higher mineralization rates (Ojanen & Minkkinen, 2020), could yield a net emissions reduction of 0.68 ± 0.026 Pg CO_2_eq. year^−1^ if all such areas were returned to intact conditions (Figure 2b). In this case, however, the benefits of a strategic restoration approach compared to the random scenario are limited because recent peatland drainage occurs predominantly in tropical regions (Appendix S1: Figure S1b) where restored peatlands tend to exhibit higher CH_4_ emissions (Feng et al., 2022).

### Optimized restoration for multiple co‐benefit objectives

**FIGURE 3:**
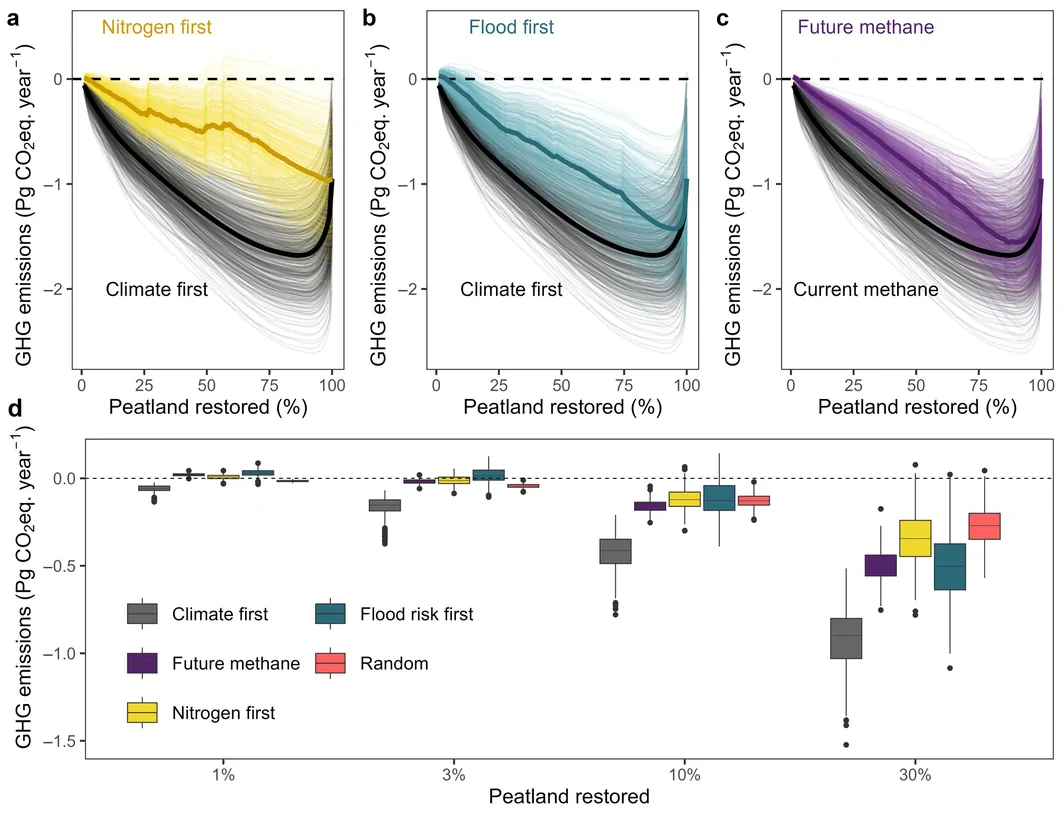
Greenhouse gas (GHG) emissions outcomes for peatland restoration strategies for multiple objectives. (a–c) Net change in GHG emissions following strategic peatland restoration in terms of CO_2_eq. (sustained‐flux global warming potential over 20 years [SGWP20]) that prioritizes emissions reductions, that is, “climate first,” versus “nitrogen first” (a), “flood first” (b), and avoiding area of high CH_4_ emissions growth thereby minimizing “future methane” emissions (c). Each line represents one outcome from the Monte Carlo simulation, and the bold lines represent scenario means and the variation in emissions at 100% restoration is due to uncertainty in emission rates as represented by Monte Carlo simulations. (d) Comparison of emission from all restoration strategies plus random restoration across varying restoration magnitudes up to 30% of global peatlands restored. The middle horizontal line in each boxplot denotes the median and the lower and upper box bounds correspond to the 25th and 75th percentiles, respectively. Boxplot whiskers extend to the most extreme data point that is less than 1.5 times the inter quartile range away from the box, beyond which outlier points are depicted with dots.

An ambitious target of 30% is often used as a goal for global conservation and restoration efforts (CBD, 2022; European Commission, 2020). Meeting such a restoration goal via strategic peatland rewetting could achieve as much as −0.90 ± 0.17 Pg CO_2_eq. year^−1^ in net emissions reductions. Meeting the same 30% restoration target while prioritizing either flood mitigation (“flood first”), or water quality improvements (using nitrogen fertilizer application as proxy, i.e., “nitrogen first”) results in net emissions of −0.50 ± 0.19 and −0.34 ± 0.14 Pg CO_2_eq. year^−1^, respectively, which is 56% and 38% of the emissions reduction potential of prioritizing GHG emissions alone (Figure 3). The difference in emissions outcome between the “flood first” strategy, and the “nitrogen first” strategies are similar to reductions achieved by the restoration of a random 10% of global peatland area (*z*‐test results: *z*_flood‐random_ = 1.5, *p*_flood‐random_ = 0.14 and *z*_nitrogen‐random_ = 2.8, *p*_nitroen‐random_ = 0.005). However, pursuing a 30% global peatland restoration target aimed toward either “flood first” or “nitrogen first” restoration would result in significantly more emissions reductions than a random approach (*z*_flood‐random_ = −24.3, *p*_flood‐random_ < 0.0001; *z*_nitrogen‐random_ = −9.1, *p*_nitrogen‐random_ < 0.0001). In general, we find that co‐benefit guided restoration does not greatly exacerbate GHG emissions, and it is possible to achieve greater emissions reductions through multi‐objective optimization approaches than through restoration at random.

**FIGURE 4:**
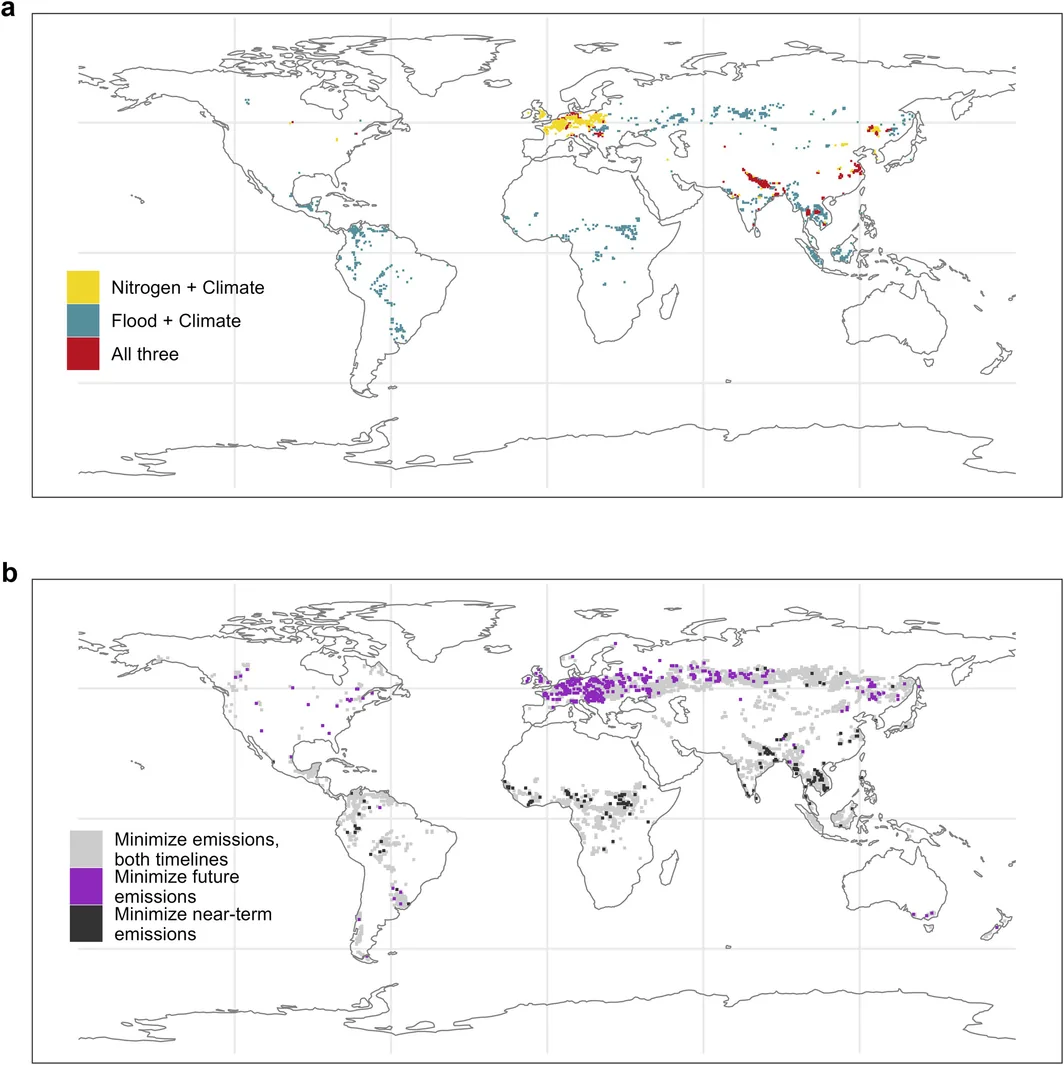
Maps of peatland restoration priority areas under different restoration objectives. (a) Map of areas where peatland restoration is most capable of achieving two or more ecosystem service objectives: water quality and climate change mitigation (yellow), flood mitigation and climate change mitigation (blue), and all three (red) (top 30th percentile for each outcome). Note that this map does not show areas with a high potential of emissions reduction that do not overlap with a high need for one of the other two ecosystem services. (b) Changes in target restoration sites when prioritizing near‐term versus future (2100) emissions reductions. Areas in purple become high priority (top 30th percentile of emissions reductions potential) when targeting reductions in future emissions while areas in dark gray are only high priority for achieving near‐term emissions reductions. Areas in light gray remain high priority for peatland restoration regardless of the timeframe used to set the emissions reduction goal. Note that pixel size has been exaggerated for visual clarity and areas with less than 1% present‐day peatland coverage or peatland loss have been masked out for visual clarity and given low opportunity for restoration.

When identifying restoration opportunities that address multiple objectives (i.e., flooding, water quality, and climate), we find three times more overlap between areas contributing to both climate and flood objectives, than for the overlap between water quality and climate goals (Figure 4a). The overlap between high flood mitigation and emissions reduction potential is, however, less prevalent in boreal regions, consistent with the spatial distribution of global flooding (Appendix S1: Figure S2c), but this has no bearing on the potential climate benefit of peatland restoration in boreal zones. Opportunities for peatland restoration with high emissions reduction potential and high nitrogen use tend to coincide in temperate climates and in the major global breadbaskets (agricultural areas of the Central United States, Europe, and Eastern China). Opportunities for restoration that meets all three objectives simultaneously exist, concentrated mainly in Northern India and Eastern China and, to a lesser extent, central Europe. Rice farming is a large driver of this pattern as wetlands converted to rice paddies have high GHG emissions, require high nitrogen inputs, and can often be found in flood plains or flood prone areas (Monfreda et al., 2008; Qian et al., 2023). Similarly, among peatlands drained post‐2010, where drainage impacts may be most reversible, there are regions capable of delivering all three ecosystem services, chiefly in Southeast Asia (Appendix S1: Figure S9).

Regions with high peatland CH_4_ emissions in the present‐day are only partly colocated with projected high emissions regions in 2100. This spatial divergence creates limited opportunities for minimizing both present and future emissions (Figure 4b) and instead presents a trade‐off between restoration for immediate versus future CH_4_ emissions reductions (Figure 3c). A strategy avoiding areas with high CH_4_ emission growth by 2100 still results in near‐term GHG emissions reductions and is significantly better than random restoration. For 30% drained peatland area restored, this strategy reduces near‐term emissions by 0.49 ± 0.088 Pg CO_2_eq. year^−1^ compared to 0.27 ± 0.11 Pg CO_2_eq. year^−1^ for random restoration (*z* = −36.9, *p* > 0.0001). Restoration scenarios minimizing future CH_4_ emissions would need to be more concentrated in temperate and boreal zones, with less emphasis on the tropics compared to a strategy for minimizing near‐term GHG emissions (Figure 4b).

## DISCUSSION

Our analysis of the GHG budgets associated with peatland restoration yields four key conclusions. First, strategic peatland rewetting can offer significant GHG emissions reductions compared to a random restoration approach. Second, there are opportunities for achieving multiple ecosystem service objectives through peatland restoration, and while these pathways might not maximize emissions reductions, they still result in net GHG reductions. Third, accounting for future CH_4_ emissions from peatlands is key to avoid long‐term climate change, but scenarios targeting present and future emission reduction only partially coincide. Finally, there is a fraction (12%) of peatlands for which restoration, even on long timescales, would likely increase net GHG emissions and where restoration for climate goals should be avoided. In these cases, emissions mitigation measures implemented might be employed to offset CH_4_ emissions from wetland restoration that, for other purposes (e.g., flood mitigation, biodiversity), is still advantageous.

We find that peatland restoration has a significant potential to mitigate climate change, consistent with findings from previous studies (Günther et al., 2020; Zou et al., 2022). Regardless of the emissions endpoint used (either “recently rewetted” or “restored to intact conditions”), CH_4_ emissions following restoration increase and, in most places, exceed the carbon sequestration capacity of restored peatlands. However, by interrupting the high CO_2_ emissions released from peatlands in their drained state, a net reduction in radiative forcing is achieved via restoration in nearly every part of the world (Figure 1). Even where restored peatlands are a source of GHGs, the avoided CO_2_ emissions from drained peatlands constitute a sizeable benefit to the climate. There are several regions where peatland restoration would result in a net increase in emissions of GHGs compared to the drained state, including parts of the southeastern United States, the Amazon River Basin, Central Africa, and Southeast Asia. Climate and land use following drainage are the primary factors that affect the GHG balance following peatland restoration (Ojanen & Minkkinen, 2020). Interestingly, both GHG emissions hotspots and areas with the largest potential emissions reductions following restoration lie predominantly in the tropics.

Through our analysis of strategic restoration, we conclude that avoiding restoration in areas that would increase radiative forcing (a positive change in the GHG balance shown in red in Figure 1b,c) is as important as targeting restoration in areas with a high potential for net emissions reductions (blue areas in Figure 1b,c, such as central and northern Europe). We find that restoration of just the top 1% of emissions hotspots (high emissions) could offset nearly all the emissions reductions achieved by restoring 10% of the world's peatlands if restored strategically for emissions reduction (Table 1). This finding points to the importance of spatial targeting to guide restoration efforts, even for relatively small restoration targets. It is important to note that this broad‐scale spatial targeting must be used in combination with site‐specific criteria (i.e., substrate type, water‐table depth) and consideration of the appropriate restoration approach, to effectively minimize GHG emissions. For peatland restoration in areas with high post‐restoration emissions that are still desirable for the delivery of other ecosystem services, additional strategies (i.e., careful attention to water‐table depth setting or substrate amendments) could be considered for managing CH_4_ emissions (Ballantine et al., 2015; Evans et al., 2021; Ury et al., 2024). Rising wetland CH_4_ production due to climate warming (Bao et al., 2023), particularly in tropical latitudes (Zhang et al., 2023), has implications for future GHG balance of restored peatlands. Our comparison of future restoration scenarios using both SGWP_20_ and SGWP_100_, highlights the tendency of SGWP_100_ to discount the importance of warming from CH_4_ emissions, due to its short atmospheric lifespan. However, the impact of future emissions cannot fully be realized without analogous projections of peatland CO_2_ and N_2_O in future climates. There is a need to explicitly include estimates of future CO_2_ fluxes (sensu Müller & Joos, 2021; Qiu et al., 2020) and to establish analogous projections for N_2_O fluxes from peatlands and wetlands to fully assess their net GHG emissions under future climate scenarios.

Flood mitigation, water quality improvements, and biodiversity support are leading drivers of wetland and peatland restoration (Zedler & Kercher, 2005). We find there is more spatial intersection between climate mitigation potential and the need for flood mitigation than water quality improvement from agricultural runoff. This is largely because cropland density is geographically concentrated whereas inland flood hazard is more well distributed globally. Our use of a flood hazard map does not represent flood exposure risk, which would instead yield overlap concentrated in areas with high population densities (Appendix S1: Figure S2c).

As for climate objectives, delivering flooding and water quality benefits require local consideration, as the reintroduction of peatlands in the landscape does not always lead to reduced flooding or improved water quality (Acreman & Holden, 2013; Koskinen et al., 2017; Nieminen et al., 2020). Peatland restoration as a natural climate solution has yet to become a mainstream practice, although peatland conservation is widely used in this way (Girkin et al., 2023). Co‐occurrence mapping, as we demonstrate in this study (Figure 4), narrows down candidate sites for restoration meeting multiple objectives. The framework we present has applications beyond peatlands and could be expanded to better understand restoration priorities for all classes of wetland. For example, coastal wetlands and peatlands have great potential for carbon sequestration and are not fully captured in this study (Bertolini & da Mosto, 2021). The CH_4_ emissions reductions achieved by restoring tidal connections in coastal wetlands demonstrate an example of a potential climate‐friendly restoration priority (Holmquist et al., 2023). Demonstrating the ability of a restoration project to deliver additional benefits in terms of climate change mitigation may help secure additional support or funding (Yi et al., 2024). Indeed, this model of benefit stacking may help to overcome some of the socioeconomic bottlenecks impeding the progress of restoration.

### Limitations

A major source of uncertainty in our analysis comes from the GHG emission rates and veracity issues inherent in global‐scale data. Data scarcity required us to collate the best available data from multiple different sources and combine rates from measured emission factors and land surface models and to use a Monte Carlo approach to estimate uncertainty. There is a pressing need for more robust GHG flux data from restored peatlands, both spatially (particularly in the tropics; Murguia‐Flores et al., 2023) and temporally (both seasonally and in terms of time since restoration as labile carbon stocks are depleted; Bianchi et al., 2021; Qiu et al., 2021). Moreover, aggregation of sparse flux data in this analysis prevented us from accounting for fine‐scale spatial and temporal factors like soil carbon, precipitation regime (Hu et al., 2024; Nisbet, 2023), and land management history that drive differences in emissions across peatlands. Our results should not be used to inform local‐scale decisions. In the future, refined emissions estimates could more explicitly include the effect of water level and its seasonal variability on the GHG balance of restored peatlands (Holmquist et al., 2023; Li et al., 2022; Pärn et al. 2023). An example of consideration of local peatland types and restoration pathways across Finland drew on GHG emissions rates informed by nutrient status of forestry‐drained peatlands and vegetation communities to develop specific recommendations for action (Laine et al., 2024). Further research that incorporates locally tuned emissions factors and wetland characteristics will enhance the ability of resource managers to put these prioritization frameworks into practice.

Some assumptions were also required to represent restoration co‐benefits. Delivering multiple ecosystem services through single restoration projects, as we assume in our multi‐objective optimization, is difficult and may be more feasible at the landscape scale rather than single sites (Hambäck et al., 2023). In future studies, additional ecosystem services, including habitat for biodiversity, fishery support, climate cooling from evaporative fluxes, and recreational and cultural services could be incorporated into a prioritization framework to further maximize social and environmental benefits from peatland restoration.

### Informing restoration action

Our analysis provides a framework for prioritizing peatland restoration that offers global context for individual interventions that can be further informed by local data and regional planning needs. While this framework does not inform decision making on the scale of individual restoration projects, it may help direct restoration spending across regional or national jurisdictions. We find that restorable peatlands with high emissions reduction potential are widely distributed. Some of the largest emissions reduction potentials are found in tropical latitudes; however, in other parts of the tropics, peatland restoration may lead to additional GHG emissions. This emphasizes the need for locally calibrated emissions estimates. This distribution of emissions reduction potential does not necessarily align with peatland restoration opportunity. The largest concentration of drained peatlands is in Europe and while not necessarily the top geographic region for emissions reduction potential, there is still tremendous climate benefit from restoring peatlands here. Recent efforts to coordinate peatland restoration in Europe with consideration for climate benefit demonstrate the potential efficacy of multinational restoration efforts (De La Haye et al., 2021).

There is still a tremendous need for research in support of guiding local restoration efforts with respect to peatland GHG emissions abatement as well as methods for restoration best management practices for minimizing emissions (Turmel‐Courchesne et al., 2023; Zou et al., 2022). Additionally, there are many social and economic barriers to peatland restoration at local levels, including the need for landowner buy‐in and the use of drained peatlands for vital economic activity that may supersede ecosystem service needs in the hierarchy of decision‐making for restoration planning (Glenk et al., 2014; Glenk & Martin‐Ortega, 2018). Our findings do, however, build on previous studies that demonstrate the urgency of peatland restoration (Günther et al., 2020; Zou et al., 2022), expanding on this body of work through restoration scenarios that identify key regions where limited funding for restoration could be directed first. Finally, the conservation of intact peatlands is key to avoiding additional emissions and must remain a priority (Girkin et al., 2023; Taillardat et al., 2020). As complex trade‐offs in emissions from restored peatlands may have limited their prominence among climate solutions, our findings help highlight the tremendous potential of peatland restoration across the world for climate and other co‐benefits.

## AUTHOR CONTRIBUTIONS

Emily A. Ury and Brian Buma conceived and designed the study. Emily A. Ury performed the data extraction, preliminary analysis and figure design. Emily A. Ury, Brian Buma, Zhen Zhang, Etienne Fluet‐Chouinard, Tianyi Sun, and Stavroula S. Sartzetakis contributed to method development and analysis of findings. Emily A. Ury wrote the first draft of the manuscript and all authors contributed to revision of the text.

## CONFLICT OF INTEREST STATEMENT

The authors declare no conflicts of interest.

## Supporting information


Appendix S1.

## Data Availability

Data used in this study are available as described in Appendix S1: Table S1. Code (Ury et al., 2026) is available in Zenodo at https://doi.org/10.5281/zenodo.21875115.
