# Supplementary material for: Strategic restoration of global inland peatlands can maximize local and global co‐benefits
Source: Ecol Appl. 2026 Sep 8;36(6):e70300. doi: 10.1002/eap.70300 (PMC13551036; doi:10.1002/eap.70300)
Supplement: Supplementary file 1 [file EAP-36-e70300-s001.pdf]

Appendix S1

Strategic restoration of global inland peatlands can maximize local and global co-benefits

Emily A. Ury, Zhen Zhang, Etienne Fluet-Chouinard, Stavroula S. Sartzetakis, Tianyi Sun, Brian Buma

Ecological Applications

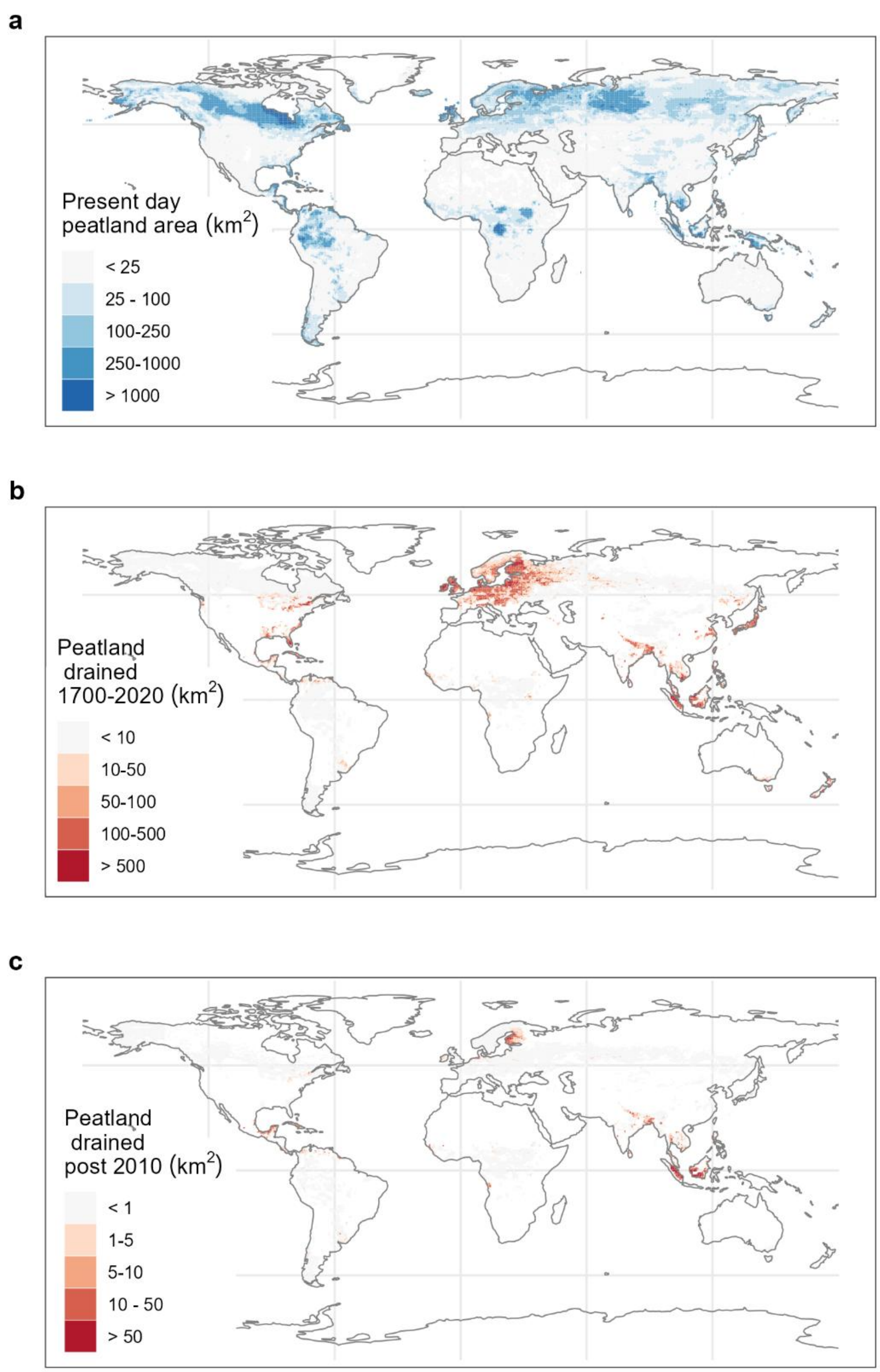

Figure S1. Global peatlands and peatland loss maps. (a) Present day peatland extent created from data presented in Melton et al. (2022). (b) Peatland area drained between 1700 and 2020, generated by calculating the fraction of peatland area drained from data presented in Fluet-Chouinard et al. (2023) using the proportion of present-day peatland area to total wetland area. (c) Peatland area drained post-2010. Note the change of scales across each panel of the plot; color breaks were assigned to enhance visual clarity.

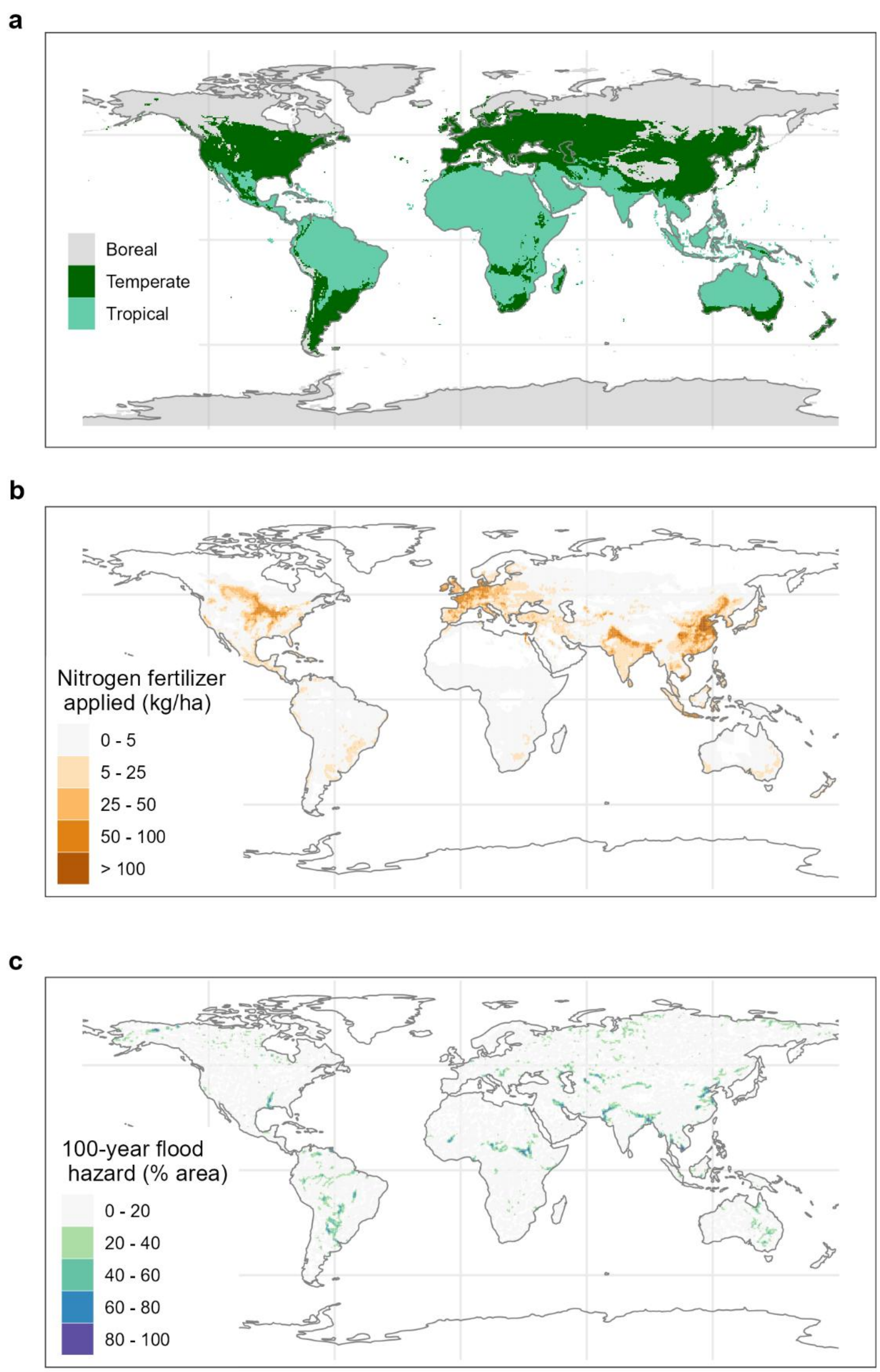

Figure S2. Auxiliary data input maps. (a) Köppen-Geiger climate zones from Beck et al. (2023) used to assign emission factors. (b) Global nitrogen fertilizer application map (data from Potter et al. 2012). (c) Flood-risk (100-year) map presented as percent of each 0.5-degree grid cell at risk (data from Dottori et al. 2016).

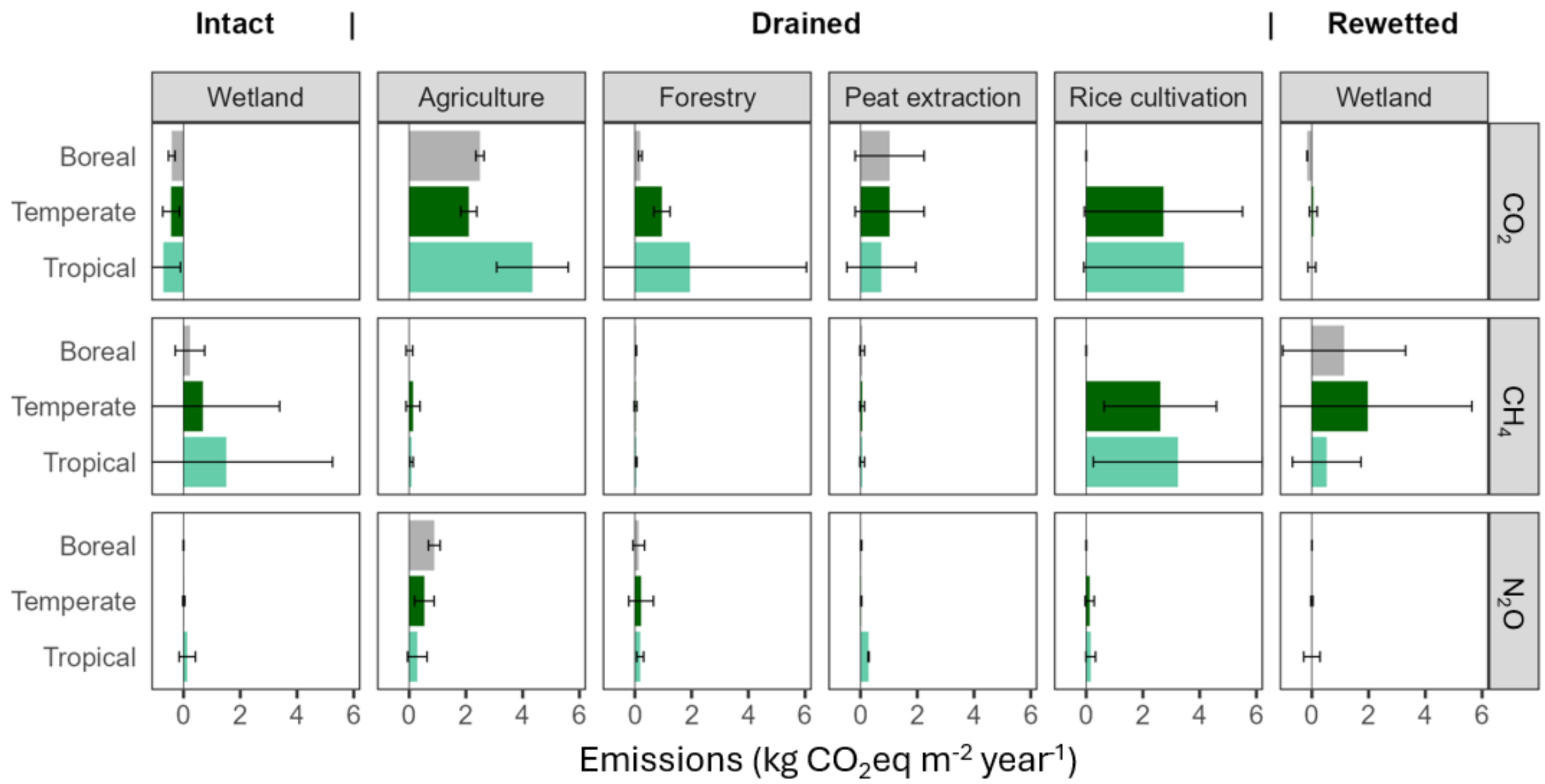

Figure S3. Mean GHG emissions factors from the IPCC (2014) by land use and climate zone. Error bars represent one standard deviation from the mean and have been truncated to improve overall readability.

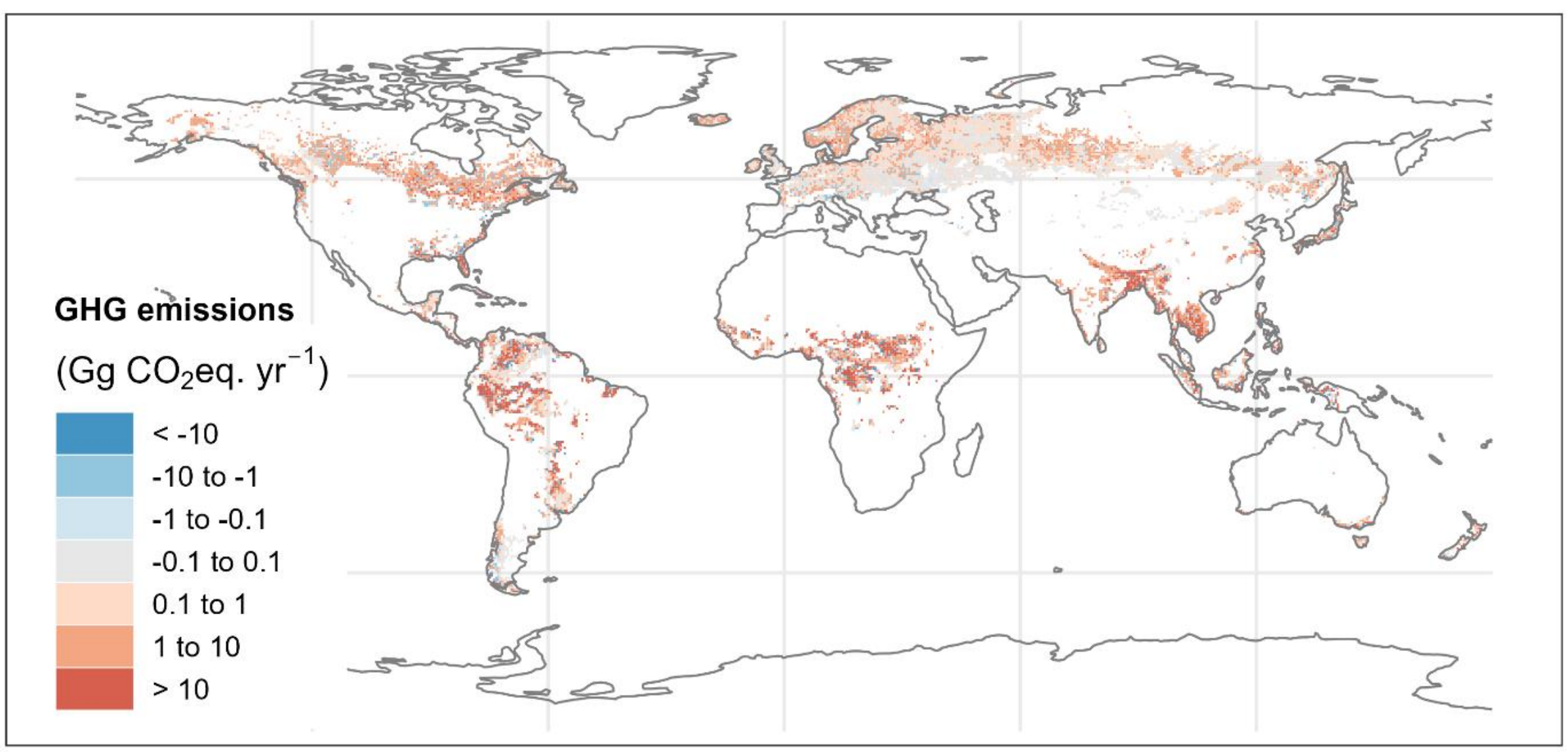

Figure S4. Difference in peatland methane emissions between the years 2099 and 2020. Positive values represent greater wetland methane emissions in the future (2099) as compared to present-day emissions (2020). The map has been masked to show only grid cells with more than 1% present-day peatland coverage for visual clarity.

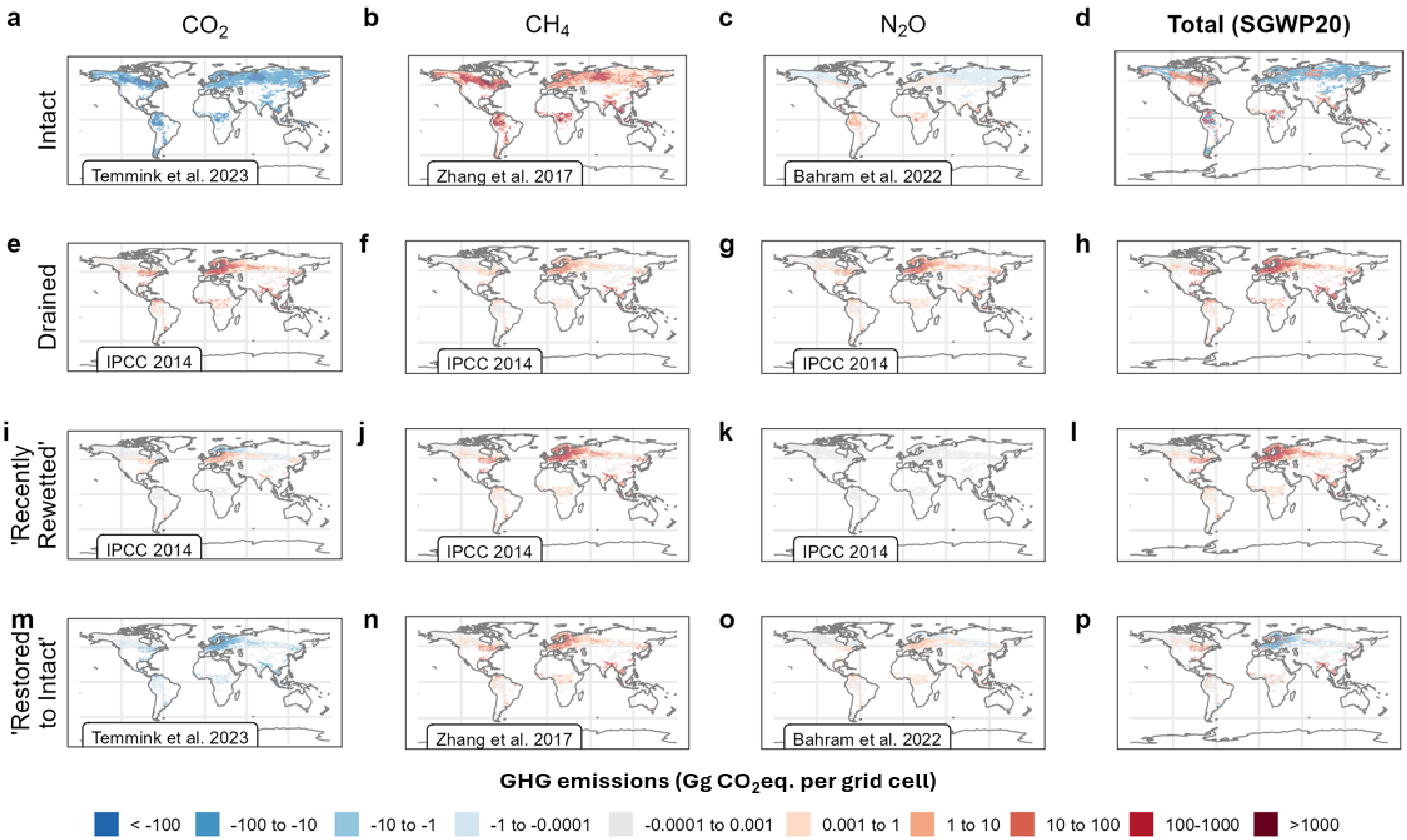

Figure S5. Greenhouse gas (CO_2, CH_4, and N_2O) emissions from intact, drained and hypothetically restored peatlands. Each map depicts by-pixel summed GHG emissions expressed in Gg CO_2eq. computed using sustained-flux Global Warming Potential over 20 years (SGWP20). (a–d) CO_2, CH_4, N_2O, and total GHG emissions for intact peatlands. (e–h) CO_2, CH_4, N_2O, and total GHG emissions for drained peatlands. (i–l) CO_2, CH_4, N_2O, and total GHG emissions potential for a scenario in which all drained peatlands were "recently rewetted". (m–p) CO_2, CH_4, N_2O, and total GHG emissions potential for a scenario in which all drained peatlands have been "restored to intact

conditions" by applying emissions factors from a-c. over areas of drained peatlands globally. Data sources are labeled on each map (the IPCC reports N_2O emissions from rewetted peatlands as not significantly different from zero). Data from the IPCC for CH_4 and N_2O emissions from drained peatlands are supplemented with data from Qian et al. 2023 for wetlands drained to rice paddies. Note color scale does not represent equal sized breaks in the data but was assigned to enhance visual clarity across all GHGs with varying emission intensity. Likewise, grid cells with less than 1% present-day peatland coverage have been masked out for visual clarity.

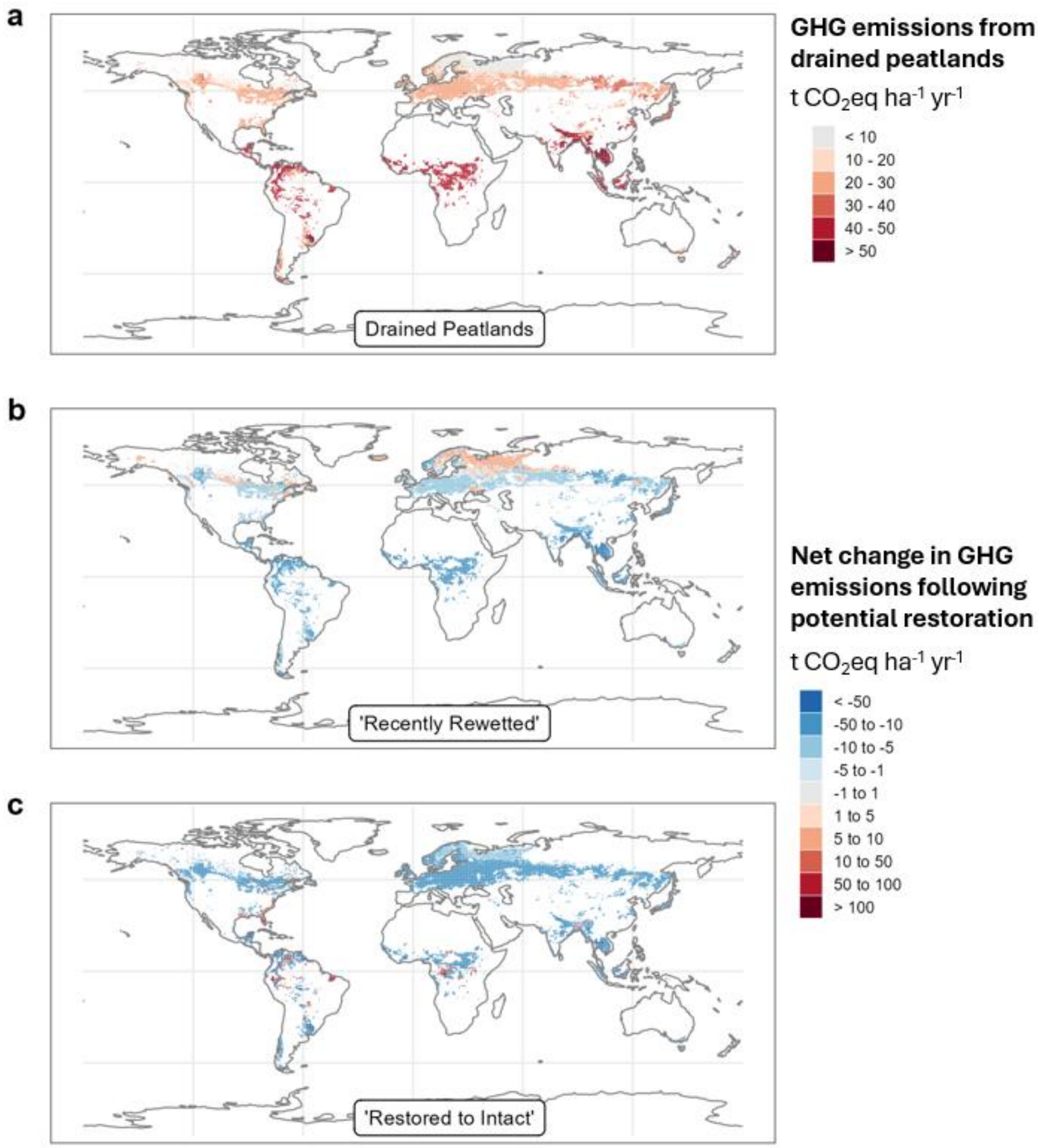

Figure S6. Area-normalized GHG emission rates from drained peatlands and net change in emission following restoration. (a) GHG emission rate from drained peatlands. (b,c) Change in emission rate following conversion of all drained peatlands to "recently rewetted" (b) or "restored to intact" (c) conditions as the final endpoints. Blue colors indicate negative emission rates (GHG sequestration), and red colors indicate positive emission rates. Emissions are expressed in metric tons (t) of CO_2eq. per hectare (ha) per year (yr) using a sustained-flux Global Warming Potential over 20 years (SGWP20) and mapped over areas of global peatland drainage. Note color scale does not represent equal sized breaks in the data and grid cells with less than 1% present-day peatland coverage have been masked out for visual clarity.

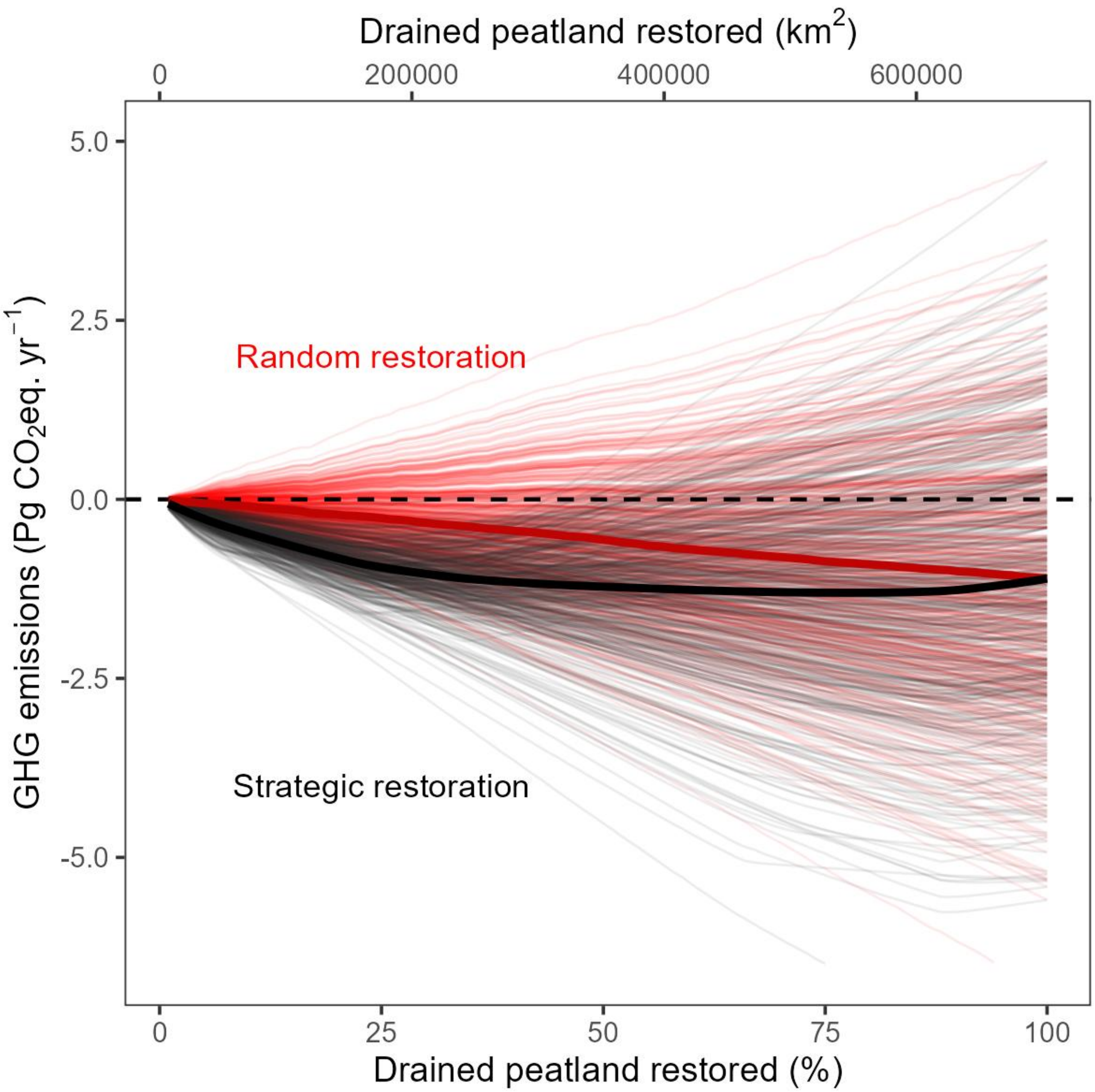

Figure S7. Net emissions potential following peatland restoration using "recently rewetted" as the endpoint for restoration**.** Net emissions across restoration magnitude in terms of CO_2eq. (SGWP$_{20}$) for both strategic (black lines) and random (red lines) restoration approaches from zero to 100% peatlands restored. Each line represents one iteration of the Monte Carlo simulation, and the bold lines represent the scenario means.

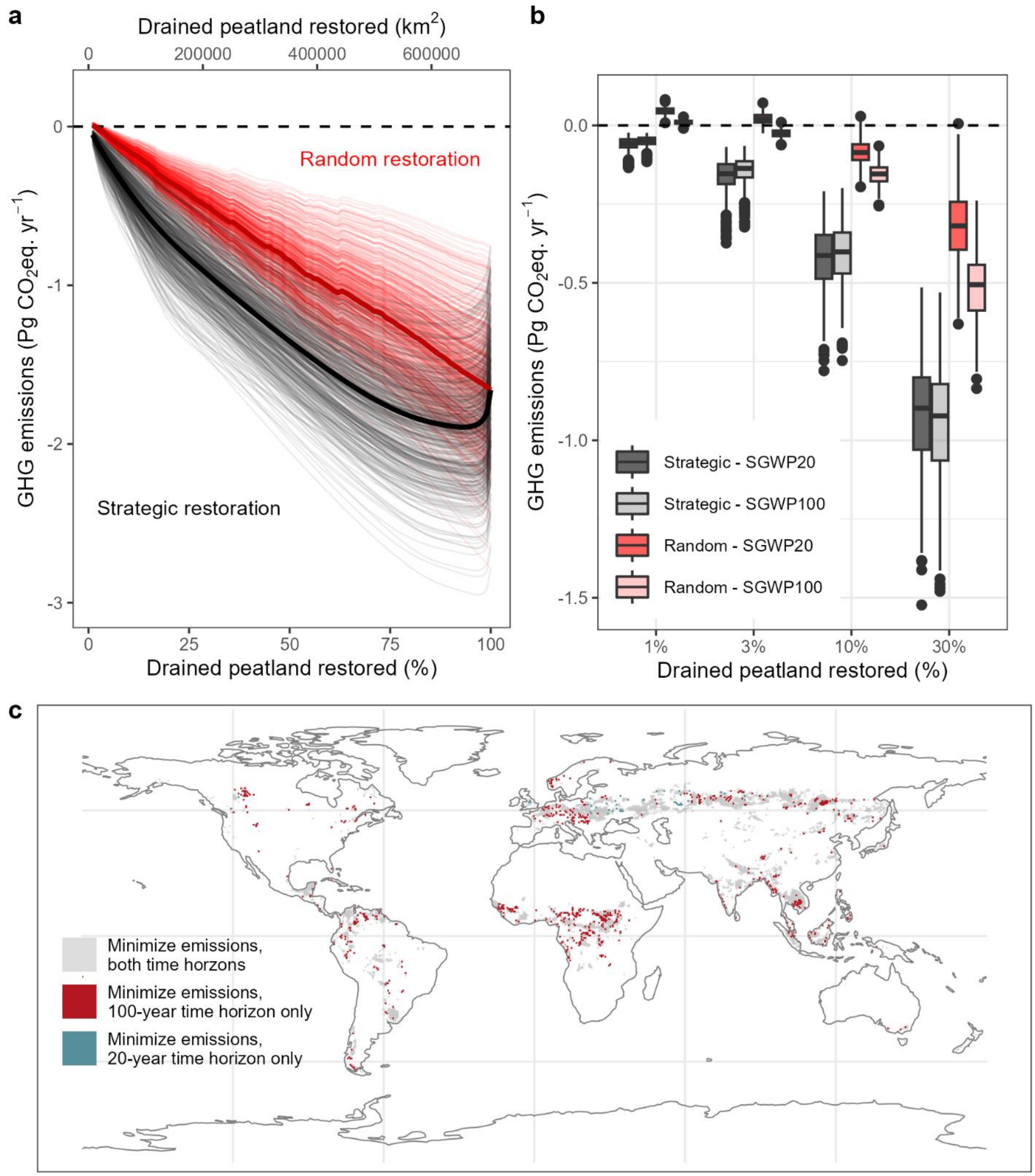

Figure S8. Strategic versus random restoration outcomes with SGWP calculated over a 100-year time horizon. (a) Net GHG emissions in terms of CO_2eq. ($SGWP_{100}$) for both strategic (black lines) and random (red lines) restoration approaches from zero to 100% peatlands restored. Each line represents one outcome from the Monte Carlo simulation and the bold lines represent the mean. (b) Comparison of emissions from strategic (gray) and random (red) restoration strategies for both 20-year (dark shades) and 100-year (light shades) time horizons across varying restoration

magnitudes up to 30% of global peatlands restored. The middle horizontal line in each boxplot denotes the median and the lower and upper box bounds correspond to the 25th and 75th percentiles, respectively. Boxplot whiskers extend to the most extreme data point that is less than 1.5 times the interquartile range away from the box, beyond which outlier points are depicted with dots. (c) Map of the top 30th percentile of climate-first restoration priority areas (lowest net emissions upon rewetting) based on either 20-year (blue) or 100-year (red) time horizons. Areas in gray are in the top 30th percentile regardless of the time horizon used. Note, that that colored pixels have been slightly enlarged and grid cells with less than 1% present day peatland coverage have been masked out for visual clarity.

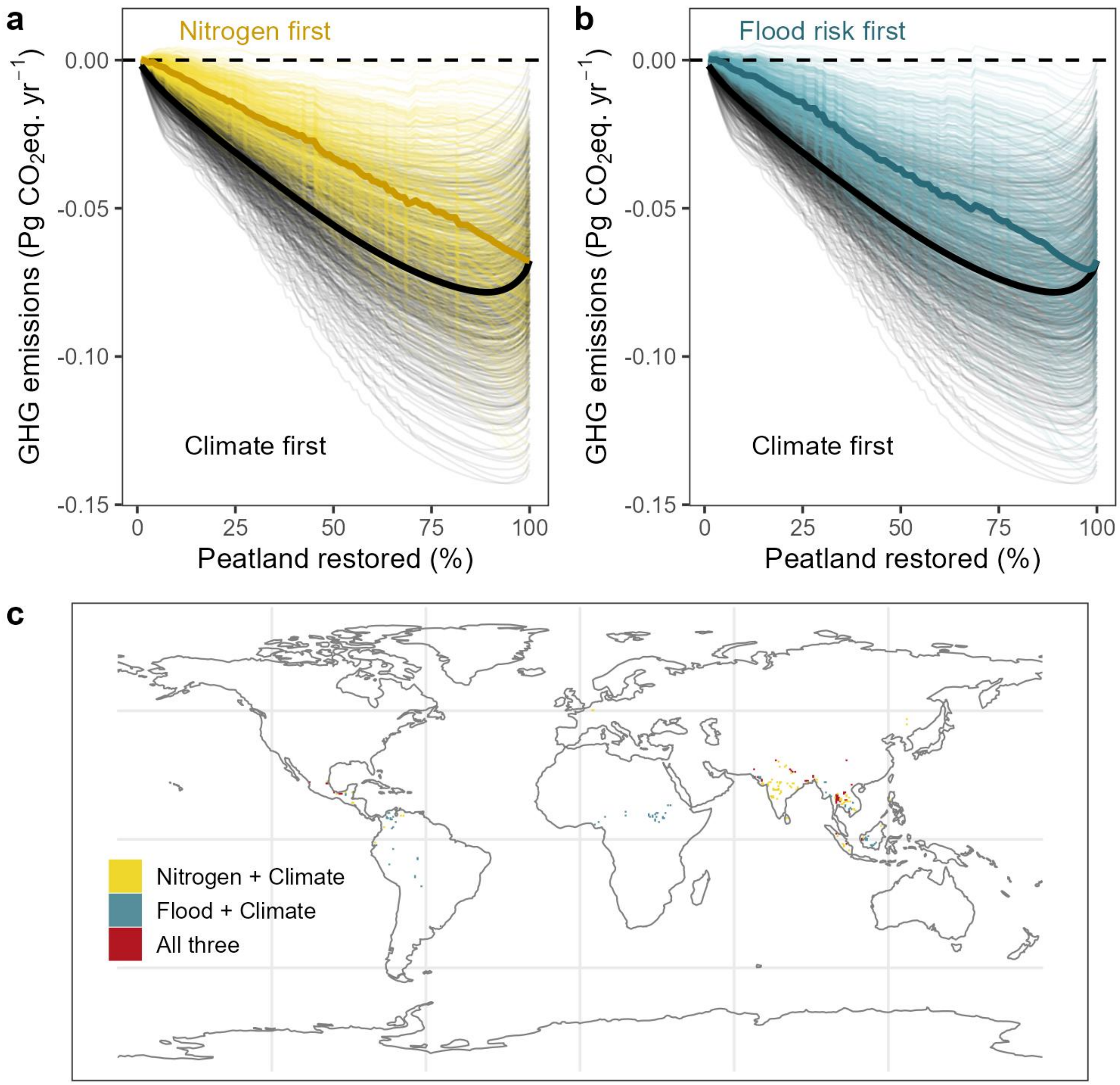

Figure S9. Comparison of restoration strategies for nitrogen and flood risk for only peatland drained post-2010. (a,b) Net emissions in terms of CO_2eq. ($SGWP_{20}$) for climate-first and nitrogen first (a) and flood-risk first (b) restoration of only peatlands drained post-2010. Each line represents one outcome from the Monte Carlo simulation and the bold lines represent the mean. (c) Map of areas where the restoration of post-2010 drained peatland is most capable of achieving two or more ecosystem service objectives: water quality and climate change mitigation (yellow), flood mitigation and climate change mitigation (blue), and all three (red). Note that colored pixels have been slightly enlarged and grid cells with less than 1% present day peatland coverage have been masked out for visual clarity.

Table S1. List of spatial data sources.

Name of database/data product	Year	Resolution	Data download links
Global wetland loss reconstruction over 1700-2020 (Fluet-Chouinard et al. 2023)	1700-2020	0.5 degree	https://doi.org/10.1038/s41586-022-05572-6 (paper) https://zenodo.org/records/7616651 (data)
Global peatland extent, Peat-ML (Melton et al. 2022)	-	5 arcmin	https://doi.org/10.5194/gmd-15-4709-2022 (paper) https://zenodo.org/records/7352284 (data)
Köppen-Geiger climate zone maps (Beck et al. 2023)	1991-2020	0.5 degree	https://doi.org/10.1038/s41597-023-02549-6 (paper) https://doi.org/10.6084/m9.figshare.c.6395666.v1 (data)
Global wetland methane emissions (Zhang et al. 2017)	2020, 2099	0.5 degree	https://doi.org/10.1073/pnas.1618765114[1] (paper)
Flood hazard map (European Commission, Joint Research Centre (2026)	-	30 arcsecond	https://doi.org/10.2905/JRC.T9279B8 (data)
Global Lakes and Wetlands Database (GLWD) – Level 3 (Lehner and Döll 2004)	-	30 second	https://www.worldwildlife.org/publications/global-lakes-and-wetlands-database-lakes-and-wetlands-grid-level-3 (data)
N fertilizer (Potter et al. 2010; Potter et al. 2012)	-	0.5 degree	https://doi.org/10.1175/2009EI288.1 (paper) https://data.nasa.gov/dataset/global-fertilizer-and-manure-version-1-nitrogen-in-manure-production (data)
Natural earth[2] – world coastlines version 4.1.0	-	110 meter	https://www.naturalearthdata.com/downloads/110m-physical-vectors/110m-coastline/ (data)

[1] Data used from this publication has made available in Ury et al. (2026) at https://doi.org/10.5281/zenodo.21875115.

[2] Natural Earth provides free vector and raster map data (naturalearthdata.com).

Table S2. Mean and standard deviation for emissions factors following regrouping.

		CO_2 Tonnes CO_2 Km^{-2} yr^{-1} Mean (standard deviation)	CH_4 Kg CH_4 Km^{-2} yr^{-1} Mean (standard deviation)	N_2O Kg N_2O Km^{-2} yr^{-1} Mean (standard deviation)
**Drained for Agriculture**				
	Boreal	2493 (147)	70 (1202)	3536 (821)
	Temperate	2099 (282)	1420 (2563)	2129 (1380)
	Tropical	4345 (1261)	858 (622)	1139 (1380)
**Drained for Forestry**				
	Boreal	189 (64)	450 (118)	537 (823)
	Temperate	953 (285)	250 (521)	880 (1734)
	Tropical	1943 (4108)	490 (281)	754 (483)
**Drained for Peat Extraction**				
	Boreal	1027 (1214)	610 (849)	94 (66)
	Temperate	1027 (1214)	610 (849)	94 (66)
	Tropical	733 (1214)	610 (849)[a]	1131 (66)
**Drained for Rice Cultivation**				
	Boreal	NA	NA	NA
	Temperate	2723 (2787)[b]	27215 (20634)	491 (644)
	Tropical	3447 (3529)	33694 (31080)	641 (674)
**Rewetted**				
	Boreal	-163 (8)	11867 (22562)	0[c]
	Temperate	50 (137)	20533 (38222)	0
	Tropical	0 (137)	5467 (12603)	0
**Intact**				
	Boreal	-415 (117)	2321 (5546)	-5 (18)
	Temperate	-438 (295)	7153 (28722)	42 (148)
	Tropical	-711 (604)	15563 (38830)	530 (1150)

[a] Assumed to be the same as temperate sites.
[b] Estimated as 79% of the CO_2 emissions from tropical rice.
[c] Emission factors for N_2O in rewetted wetlands do not significantly differ from zero (IPCC 2014).

Table S3. Emission factors from original sources and regrouping assignments.

Source	Loca-tion	Status	Source land use	Sub type	Source climate zone	GHG	Units	Emission Factor (mean)	CI low	CI high	n	sd	Assigned land use	Assigned climate zone	Land use combinations from Fluet-Chouinard (2023)
IPCC Chapter 2	Table 2.1	Drained	Forest including shrubland	Nutrient-poor	Boreal	CO2	tonnes CO2-C ha-1 yr-1	0.37	-0.11	0.84	63		Forestry	Boreal	Forestry
IPCC Chapter 2	Table 2.1	Drained	Forest	Nutrient-poor	Boreal	CO2	tonnes CO2-C ha-1 yr-1	0.25	-0.23	0.73	59		Forestry	Boreal	Forestry
IPCC Chapter 2	Table 2.1	Drained	Forest	Nutrient-rich	Boreal	CO2	tonnes CO2-C ha-1 yr-1	0.93	0.54	1.3	62		Forestry	Boreal	Forestry
IPCC Chapter 2	Table 2.1	Drained	Forest		Temperate	CO2	tonnes CO2-C ha-1 yr-1	2.6	2	3.3	8		Forestry	Temperate	Forestry
IPCC Chapter 2	Table 2.1	Drained	Forest and cleared shrubland		Tropical	CO2	tonnes CO2-C ha-1 yr-1	5.3	-0.7	9.5	21		Forestry	Tropical	Forestry
IPCC Chapter 2	Table 2.1	Drained	Plantations	long rotation	Tropical	CO2	tonnes CO2-C ha-1 yr-1	15	10	21			Agriculture	Tropical	Cropland + Pasture + Wetland Cultivation
IPCC Chapter 2	Table 2.1	Drained	Plantations	short rotation	Tropical	CO2	tonnes CO2-C ha-1 yr-1	20	16	24	13		Agriculture	Tropical	Cropland + Pasture + Wetland Cultivation
IPCC Chapter 2	Table 2.1	Drained	Plantations	oil palm	Tropical	CO2	tonnes CO2-C ha-1 yr-1	11	5.6	17	10		Agriculture	Tropical	Cropland + Pasture + Wetland Cultivation
IPCC Chapter 2	Table 2.1	Drained	Plantations	shallow-drained	Tropical	CO2	tonnes CO2-C ha-1 yr-1	1.5	-2.3	5.4	5		Agriculture	Tropical	Cropland + Pasture + Wetland Cultivation
IPCC Chapter 2	Table 2.1	Drained	Cropland		Boreal/ Temperate	CO2	tonnes CO2-C ha-1 yr-1	7.9	6.5	9.4	39		Agriculture	Boreal	Cropland + Pasture + Wetland Cultivation
IPCC Chapter 2	Table 2.1	Drained	Cropland		Boreal/ Temperate	CO2	tonnes CO2-C ha-1 yr-1	7.9	6.5	9.4	39		Agriculture	Temperate	Cropland + Pasture + Wetland Cultivation
IPCC Chapter 2	Table 2.1	Drained	Cropland	fallow	Tropical	CO2	tonnes CO2-C ha-1 yr-1	14	6.6	26	10		Agriculture	Tropical	Cropland + Pasture + Wetland Cultivation
IPCC Chapter 2	Table 2.1	Drained	Cropland	paddy rice	Tropical	CO2	tonnes CO2-C ha-1 yr-1	9.4	-0.2	20	6		Rice	Tropical	Rice
IPCC Chapter 2	Table 2.1	Drained	Grassland		Boreal	CO2	tonnes CO2-C ha-1 yr-1	5.7	2.9	8.6	8		Agriculture	Boreal	Cropland + Pasture + Wetland Cultivation
IPCC Chapter 2	Table 2.1	Drained	Grassland	nutrient-poor	Temperate	CO2	tonnes CO2-C ha-1 yr-1	5.3	3.7	6.9	7		Agriculture	Temperate	Cropland + Pasture + Wetland Cultivation
IPCC Chapter 2	Table 2.1	Drained	Grassland	nutrient-rich	Temperate	CO2	tonnes CO2-C ha-1 yr-1	6.1	5	7.3	39		Agriculture	Temperate	Cropland + Pasture + Wetland Cultivation

IPCC Chapter 2	Table 2.1	Drained	Grassland	shallow-drained, nutrient rich	Temperate	CO2	tonnes CO2-C ha-1 yr-1	3.6	1.8	5.4	13		Agriculture	Temperate	Cropland + Pasture + Wetland Cultivation
IPCC Chapter 2	Table 2.1	Drained	Grassland		Tropical	CO2	tonnes CO2-C ha-1 yr-1	9.6	4.5	17			Agriculture	Tropical	Cropland + Pasture + Wetland Cultivation
IPCC Chapter 2	Table 2.1	Drained	Peatland manage for extraction		Boreal/ Temperate	CO2	tonnes CO2-C ha-1 yr-1	2.8	1.1	4.2	20		Peat extraction	Boreal	Peat extraction
IPCC Chapter 2	Table 2.1	Drained	Peatland manage for extraction		Boreal/ Temperate	CO2	tonnes CO2-C ha-1 yr-1	2.8	1.1	4.2	20		Peat extraction	Temperate	Peat extraction
IPCC Chapter 2	Table 2.1	Drained	Peatland manage for extraction		Tropical	CO2	tonnes CO2-C ha-1 yr-1	2	0.06	7			Peat extraction	Tropical	Peat extraction
IPCC Chapter 2	Table 2.3	Drained	Forest	Nutrient-poor	Boreal	CH4	kg CH4 ha-1 yr-1	7	2.9	11	47		Forestry	Boreal	Forestry
IPCC Chapter 2	Table 2.3	Drained	Forest	Nutrient-rich	Boreal	CH4	kg CH4 ha-1 yr-1	2	-1.6	5.5	83		Forestry	Boreal	Forestry
IPCC Chapter 2	Table 2.3	Drained	Forest		Temperate	CH4	kg CH4 ha-1 yr-1	2.5	-0.6	5.7	13		Forestry	Temperate	Forestry
IPCC Chapter 2	Table 2.3	Drained	Forest and cleared shrubland		Tropical	CH4	kg CH4 ha-1 yr-1	4.9	2.3	7.5	7		Forestry	Tropical	Forestry
IPCC Chapter 2	Table 2.3	Drained	Plantations		Tropical/ subtropical	CH4	kg CH4 ha-1 yr-1	2.7	-0.9	6.3	5		Agriculture	Tropical	Cropland + Pasture + Wetland Cultivation
IPCC Chapter 2	Table 2.3	Drained	Plantations	oil palm	Tropical/ subtropical	CH4	kg CH4 ha-1 yr-1	0	0	0	1		Agriculture	Tropical	Cropland + Pasture + Wetland Cultivation
IPCC Chapter 2	Table 2.3	Drained	Plantations	sago palm	Tropical/ subtropical	CH4	kg CH4 ha-1 yr-1	26.2	7.2	45.3	6		Agriculture	Tropical	Cropland + Pasture + Wetland Cultivation
IPCC Chapter 2	Table 2.3	Drained	Cropland		Boreal/ Temperate	CH4	kg CH4 ha-1 yr-1	0	-2.8	2.8	38		Agriculture	Boreal	Cropland + Pasture + Wetland Cultivation
IPCC Chapter 2	Table 2.3	Drained	Cropland		Boreal/ Temperate	CH4	kg CH4 ha-1 yr-1	0	-2.8	2.8	38		Agriculture	Temperate	Cropland + Pasture + Wetland Cultivation
IPCC Chapter 2	Table 2.3	Drained	Cropland		Tropical/ subtropical	CH4	kg CH4 ha-1 yr-1	7	0.3	13.7	5		Agriculture	Tropical	Cropland + Pasture + Wetland Cultivation
IPCC Chapter 2	Table 2.3	Drained	Grassland		Boreal	CH4	kg CH4 ha-1 yr-1	1.4	-1.6	45	12		Agriculture	Boreal	Cropland + Pasture + Wetland Cultivation
IPCC Chapter 2	Table 2.3	Drained	Grassland	nutrient-poor	Temperate	CH4	kg CH4 ha-1 yr-1	1.8	0.72	2.9	9		Agriculture	Temperate	Cropland + Pasture + Wetland Cultivation

IPCC Chapter 2	Table 2.3	Drained	Grassland	deep-drained, nutrient-rich	Temperate	CH4	kg CH4 ha-1 yr-1	16	2.4	29	44		Agriculture	Temperate	Cropland + Pasture + Wetland Cultivation
IPCC Chapter 2	Table 2.3	Drained	Grassland	shallow-drained, nutrient rich	Temperate	CH4	kg CH4 ha-1 yr-1	39	-2.9	81	16		Agriculture	Temperate	Cropland + Pasture + Wetland Cultivation
IPCC Chapter 2	Table 2.3	Drained	Grassland		Tropical/ subtropical	CH4	kg CH4 ha-1 yr-1	7	0.3	13.7	5		Agriculture	Tropical	Cropland + Pasture + Wetland Cultivation
IPCC Chapter 2	Table 2.3	Drained	Peatland manage for extraction		Boreal/ Temperate	CH4	kg CH4 ha-1 yr-1	6.1	1.6	11	15		Peat extraction	Boreal	Peat extraction
IPCC Chapter 2	Table 2.3	Drained	Peatland manage for extraction		Tropical	CH4	kg CH4 ha-1 yr-1	6.1	1.6	11	15		Peat extraction	Tropical	Peat extraction
IPCC Chapter 2	Table 2.3	Drained	Peatland manage for extraction		Boreal/ Temperate	CH4	kg CH4 ha-1 yr-1	6.1	1.6	11	15		Peat extraction	Temperate	Peat extraction
IPCC Chapter 2	Table 2.5	Drained	Forest	Nutrient-poor	Boreal	N2O	kg N2O-N ha-1 yr-1	0.22	0.15	0.28	43		Forestry	Boreal	Forestry
IPCC Chapter 2	Table 2.5	Drained	Forest	Nutrient-rich	Boreal	N2O	kg N2O-N ha-1 yr-1	3.2	1.9	4.5	75		Forestry	Boreal	Forestry
IPCC Chapter 2	Table 2.5	Drained	Forest		Temperate	N2O	kg N2O-N ha-1 yr-1	2.8	-0.57	6.1	13		Forestry	Temperate	Forestry
IPCC Chapter 2	Table 2.5	Drained	Forest and cleared shrubland		Tropical/ Subtropical	N2O	kg N2O-N ha-1 yr-1	2.4	1.3	3.5	10		Forestry	Tropical	Forestry
IPCC Chapter 2	Table 2.5	Drained	Plantations	oil palm	Tropical/ Subtropical	N2O	kg N2O-N ha-1 yr-1	1.2			1		Agriculture	Tropical	Cropland + Pasture + Wetland Cultivation
IPCC Chapter 2	Table 2.5	Drained	Plantations	sago	Tropical/ Subtropical	N2O	kg N2O-N ha-1 yr-1	3.3			1		Agriculture	Tropical	Cropland + Pasture + Wetland Cultivation
IPCC Chapter 2	Table 2.5	Drained	Cropland		Boreal/ Temperate	N2O	kg N2O-N ha-1 yr-1	13	8.2	18	36		Agriculture	Boreal	Cropland + Pasture + Wetland Cultivation
IPCC Chapter 2	Table 2.5	Drained	Cropland		Boreal/ Temperate	N2O	kg N2O-N ha-1 yr-1	13	8.2	18	36		Agriculture	Temperate	Cropland + Pasture + Wetland Cultivation
IPCC Chapter 2	Table 2.5	Drained	Cropland	except rice	Tropical/ Subtropical	N2O	kg N2O-N ha-1 yr-1	5	2.3	7.7	8		Agriculture	Tropical	Cropland + Pasture + Wetland Cultivation
IPCC Chapter 2	Table 2.5	Drained	Grassland		Boreal	N2O	kg N2O-N ha-1 yr-1	9.5	4.6	14	16		Agriculture	Boreal	Cropland + Pasture + Wetland Cultivation

IPCC Chapter 2	Table 2.5	Drained	Grassland	nutrient-poor	Temperate	N2O	kg N2O-N ha-1 yr-1	4.3	1.9	6.8	7		Agriculture	Temperate	Cropland + Pasture + Wetland Cultivation
IPCC Chapter 2	Table 2.5	Drained	Grassland	deep-drained, nutrient-rich	Temperate	N2O	kg N2O-N ha-1 yr-1	8.2	4.9	11	47		Agriculture	Temperate	Cropland + Pasture + Wetland Cultivation
IPCC Chapter 2	Table 2.5	Drained	Grassland	shallow-drained, nutrient rich	Temperate	N2O	kg N2O-N ha-1 yr-1	1.6	0.56	2.7	13		Agriculture	Temperate	Cropland + Pasture + Wetland Cultivation
IPCC Chapter 2	Table 2.5	Drained	Grassland		Tropical/ Subtropical	N2O	kg N2O-N ha-1 yr-1	5	2.3	7.7	8		Agriculture	Tropical	Cropland + Pasture + Wetland Cultivation
IPCC Chapter 2	Table 2.5	Drained	Peatland manage for extraction		Boreal/ Temperate	N2O	kg N2O-N ha-1 yr-1	0.3	-0.03	0.64	4		Peat extraction	Boreal	Peat extraction
IPCC Chapter 2	Table 2.5	Drained	Peatland manage for extraction		Boreal/ Temperate	N2O	kg N2O-N ha-1 yr-1	0.3	-0.03	0.64	4		Peat extraction	Temperate	Peat extraction
IPCC Chapter 2	Table 2.5	Drained	Peatland manage for extraction		Tropical/ Subtropical	N2O	kg N2O-N ha-1 yr-1	3.6	0.2	5			Peat extraction	Tropical	Peat extraction
IPCC Chapter 3	Table 3.1	Rewetted	Rewetted	nutrient poor	Boreal	CO2	tonnes CO2-C ha-1 yr-1	-0.34	-0.59	-0.09	26		Rewetted	Boreal	
IPCC Chapter 3	Table 3.1	Rewetted	Rewetted	nutrient rich	Boreal	CO2	tonnes CO2-C ha-1 yr-1	-0.55	-0.77	-0.34	39		Rewetted	Boreal	
IPCC Chapter 3	Table 3.1	Rewetted	Rewetted	nutrient poor	Temperate	CO2	tonnes CO2-C ha-1 yr-1	-0.23	-0.64	0.18	43		Rewetted	Temperate	
IPCC Chapter 3	Table 3.1	Rewetted	Rewetted	nutrient rich	Temperate	CO2	tonnes CO2-C ha-1 yr-1	0.5	-0.71	1.71	15		Rewetted	Temperate	
IPCC Chapter 3	Table 3.1	Rewetted	Rewetted		Tropical	CO2	tonnes CO2-C ha-1 yr-1	0					Rewetted	Tropical	
IPCC Chapter 3	Table 3.3	Rewetted	Rewetted	nutrient poor	Boreal	CH4	kg CH4-C ha-1 yr-1	41	0.5	246	39		Rewetted	Boreal	
IPCC Chapter 3	Table 3.3	Rewetted	Rewetted	nutrient rich	Boreal	CH4	kg CH4-C ha-1 yr-1	137	0	493	35		Rewetted	Boreal	
IPCC Chapter 3	Table 3.3	Rewetted	Rewetted	nutrient poor	Temperate	CH4	kg CH4-C ha-1 yr-1	92	3	445	42		Rewetted	Temperate	
IPCC Chapter 3	Table 3.3	Rewetted	Rewetted	nutrient rich	Temperate	CH4	kg CH4-C ha-1 yr-1	216	0	856	37		Rewetted	Temperate	
IPCC Chapter 3	Table 3.3	Rewetted	Rewetted		Tropical	CH4	kg CH4-C ha-1 yr-1	41	7	134	11		Rewetted	Tropical	

IPCC Chapter 3	Table 3.3	Rewetted	Rewetted		Boreal	N2O	kg N2O-N ha-1 yr-1	0					Rewetted	Boreal	
IPCC Chapter 3	Table 3.3	Rewetted	Rewetted		Temperate	N2O	kg N2O-N ha-1 yr-1	0					Rewetted	Temperate	
IPCC Chapter 3	Table 3.3	Rewetted	Rewetted		Tropical	N2O	kg N2O-N ha-1 yr-1	0					Rewetted	Tropical	
Qian 2023	Table S1	Drained	Rice		Temperate	CH4	kg CH4 ha-1 yr-1	272.1			225	206.3	Rice	Temperate	Rice
Qian 2023	Table S1	Drained	Rice		Tropical	CH4	kg CH4 ha-1 yr-1	336.9			44	310.8	Rice	Tropical	Rice
Qian 2023	Table S1	Drained	Rice		Temperate	N2O	kg N2O ha-1 yr-1	1.56			165	2.05	Rice	Temperate	Rice
Qian 2023	Table S1	Drained	Rice		Tropical	N2O	kg N2O ha-1 yr-1	2.04			35	2.14	Rice	Tropical	Rice
Temmink 2022	Figure 1C	Intact	Wetland		Boreal	CO2	tonnes CO2-C ha-1 yr-1	-1.133				0.3196	Wetland	Boreal	Natural wetland 2020
Temmink 2022	Figure 1C	Intact	Wetland		Temperate	CO2	tonnes CO2-C ha-1 yr-1	-1.195				0.8054	Wetland	Temperate	Natural wetland 2020
Temmink 2022	Figure 1C	Intact	Wetland		Tropical	CO2	tonnes CO2-C ha-1 yr-1	-1.939				1.6479	Wetland	Tropical	Natural wetland 2020
Bahram 2022	Table S1	Intact	Wetland		Boreal	N2O	kg N2O-N ha-1 yr-1	-0.017				0.0587	Wetland	Boreal	Natural wetland 2020
Bahram 2022	Table S1	Intact	Wetland		Temperate	N2O	kg N2O-N ha-1 yr-1	0.135				0.472	Wetland	Temperate	Natural wetland 2020
Bahram 2022	Table S1	Intact	Wetland		Tropical	N2O	kg N2O-N ha-1 yr-1	1.686				3.658	Wetland	Tropical	Natural wetland 2020
Zhang 2017		Intact	Wetland		Boreal	CH_4	kg CH4 km-2 yr-1	2364				5397	Wetland	Boreal	Natural wetland 2020
Zhang 2017		Intact	Wetland		Tropical	CH_4	kg CH4 km-2 yr-1	7112				28163	Wetland	Temperate	Natural wetland 2020
Zhang 2017		Intact	Wetland		Temperate	CH4	kg CH4 km-2 yr-1	15720				38993	Wetland	Tropical	Natural wetland 2020

Note that CH4 emission from intact wetlands are from Zheng et al. and are a spatially explicit modeled data product and the values shown here are the mean for each climate zone.

References

Bahram, M., M. Espenberg, J. Pärn, L. Lehtovirta-Morley, S. Anslan, K. Kasak, U. Kõljalg, J. Liira, M. Maddison, M. Moora, Ü. Niinemets, M. Öpik, M. Pärtel, K. Soosaar, M. Zobel, F. Hildebrand, L. Tedersoo, and Ü. Mander. 2022. Structure and function of the soil microbiome underlying N2O emissions from global wetlands. Nature Communications 13:1430.

Beck, H. E., T. R. McVicar, N. Vergopolan, A. Berg, N. J. Lutsko, A. Dufour, Z. Zeng, X. Jiang, A. I. J. M. van Dijk, and D. G. Miralles. 2023. High-resolution (1 km) Köppen-Geiger maps for 1901-2099 based on constrained CMIP6 projections. Scientific Data 10:724.

Dottori, F., L. Alfieri, P. Salamon, A. Bianchi, L. Feyen, and F. Hirpa. 2016. Flood hazard map of the World - 100-year return period. European Commission, Joint Research Centre (JRC) [Dataset]. European Commission, Joint Research Centre (JRC).

European Commission, Joint Research Centre. 2026. GloFAS: flood hazard map (100 year return period). [Dataset] doi: 10.2905/JRC.T9279B8

Fluet-Chouinard, E., B. D. Stocker, Z. Zhang, A. Malhotra, J. R. Melton, B. Poulter, J. O. Kaplan, K. K. Goldewijk, S. Siebert, T. Minayeva, G. Hugelius, H. Joosten, A. Barthelmes, C. Prigent, F. Aires, A. M. Hoyt, N. Davidson, C. M. Finlayson, B. Lehner, R. B. Jackson, and P. B. McIntyre. 2023. Extensive global wetland loss over the past three centuries. Nature 614:281–286.

Intergovernmental Panel on Climate Change (IPCC). 2014. 2013 Supplement to the 2006 IPCC Guidelines for National Greenhouse Gas Inventories: Wetlands. Page (T. Hiraishi, T. Krug, K. Tanabe, N. Srivastava, B. Jamsranjav, M. Fukuda, and T. Troxler, Eds.). IPCC, Geneva, Switzerland.

Lehner, B., and P. Döll. 2004. Development and validation of a global database of lakes, reservoirs and wetlands. Journal of Hydrology. 296/1-4:1-22.

Melton, J. R., E. Chan, K. Millard, M. Fortier, R. S. Winton, J. M. Martín-López, H. Cadillo-Quiroz, D. Kidd, and L. V. Verchot. 2022. A map of global peatland extent created using machine learning (Peat-ML). Geoscientific Model Development 15:4709–4738.

Potter, P., N. Ramankutty, E. M. Bennett, and S. D. Donner. 2010. Characterizing the Spatial Patterns of Global Fertilizer Application and Manure Production. Earth Interactions 14:1–22.

Potter, P., N. Ramankutty, E. M. Bennett, and S. D. Donner. 2012. Global Fertilizer and Manure, Version 1: Nitrogen Fertilizer Application. Palisades, New York.

Qian, H., X. Zhu, S. Huang, B. Linquist, Y. Kuzyakov, R. Wassmann, K. Minamikawa, M. Martinez-Eixarch, X. Yan, F. Zhou, B. O. Sander, W. Zhang, Z. Shang, J. Zou, X. Zheng, G. Li, Z. Liu, S. Wang, Y. Ding, K. J. van Groenigen, and Y. Jiang. 2023. Greenhouse gas emissions and mitigation in rice agriculture. Nature Reviews Earth & Environment 4:716–732.

Temmink, R. J. M., L. P. M. Lamers, C. Angelini, T. J. Bouma, C. Fritz, J. van de Koppel, R. Lexmond, M. Rietkerk, B. R. Silliman, H. Joosten, and T. van der Heide. 2022. Recovering wetland biogeomorphic feedbacks to restore the world’s biotic carbon hotspots. Science 376:eabn1479.

Ury, E., Z. Zhang, E. Fluet-Chouinard, S. Sartzetakis, T. Sun, and B. Buma. 2026. Analysis code for "Strategic restoration of global inland peatlands can maximize local and global co-benefits". In Ecological Applications. Zenodo. [Software] https://doi.org/10.5281/zenodo.21875115

Zhang, Z., N. E. Zimmermann, A. Stenke, X. Li, E. L. Hodson, G. Zhu, C. Huang, and B. Poulter. 2017. Emerging role of wetland methane emissions in driving 21st century climate change. Proceedings of the National Academy of Sciences of the United States of America 114:9647–9652.
